# Selective vulnerability of the aging cholinergic system to amyloid pathology revealed by induced APP overexpression

**DOI:** 10.1186/s12974-025-03682-2

**Published:** 2026-01-07

**Authors:** Kan Xie, Devon Ryan, Susanne Schröder, Lena Freund, Stefan Bonn, Yu Zhou, Dan Ehninger

**Affiliations:** 1 Translational Biogerontology Lab, German Center for Neurodegenerative Diseases (DZNE), Venusberg-Campus 1/99, Bonn, 53127 Germany; 2 Institute of Medical Systems Bioinformatics, Center for Biomedical AI (bAIome), Center for Molecular Neurobiology (ZMNH), Center for Translational Immunology (HCTI), German Center for Child and Youth Health (DZKJ), University Medical Center Hamburg-Eppendorf, Hamburg, 20246 Germany; 3 School of Life Sciences and Health, University of Health and Rehabilitation Sciences, Qingdao, Shandong 266021 China; 4 Present address: Genedata AG, Margarethenstr. 38, Basel, 4053 Switzerland; 5 Present address: Fraunhofer Institute for Molecular Biology and Applied Ecology IME, Forckenbeckstr. 6, Aachen, 52074 Germany

**Keywords:** Alzheimer´s disease, Aβ, Aging, Cholinergic system, Neurodegeneration

## Abstract

Alzheimer’s disease (AD) is a progressive neurodegenerative disorder characterized by amyloid beta (Aβ) accumulation, tau pathology, and cognitive decline, with aging as the primary risk factor. To investigate whether age influences susceptibility to Aβ toxicity, we used a tetracycline-inducible mouse model expressing a mutant human APP transgene (APPSweInd) and initiated expression during either mid-age (6–18 months) or old age (12–24 months). After one year of transgene activation, we assessed behavior, amyloid pathology, inflammation, autophagy, and brain gene expression compared to age-matched controls. Although APP expression, Aβ deposition, inflammatory markers, and autophagic flux were comparable between age groups, aged APP-expressing mice displayed cognitive impairments, hyperactivity, and motor deficits that were absent in their younger counterparts. Transcriptomic analysis revealed selective downregulation of cholinergic system genes specifically in the aged APP-induced group, validated at RNA and protein levels. No changes were observed in markers of other neuronal cell types, indicating a targeted cholinergic vulnerability. These findings suggest that age enhances the brain’s susceptibility to Aβ toxicity, particularly affecting the cholinergic system, rather than amplifying amyloid burden itself. This inducible model provides a relevant platform to study the interaction between aging and Aβ pathology and may help identify age-related factors contributing to AD progression.

**Supplementary Information:**

The online version contains supplementary material available at 10.1186/s12974-025-03682-2.

## Introduction

Alzheimer’s disease (AD) is the most common form of dementia in the elderly, affecting over 50 million people worldwide [1, 2]. In addition to behavioral changes and progressive cognitive decline, AD is histopathologically characterized by two key features: the extracellular accumulation of senile plaques and the intracellular formation of neurofibrillary tangles (NFTs) [2–4]. Senile plaques are primarily composed of aggregated amyloid beta (Aβ) peptides, while NFTs consist of abnormally phosphorylated tau protein [2, 5]. Other pathological hallmarks include chronic neuroinflammation [6], synaptic loss [7], and neuronal degeneration (e.g. cholinergic neurons) in the brain [8, 9].

Aging is the most significant risk factor for AD [2, 5]. AD prevalence doubles approximately every five years between the ages of 50 and 80, after which the rate of increase slows due to the already high prevalence among the elderly [10]. Most cases are sporadic and diagnosed after age 65, classified as late-onset AD (LOAD) [1, 2]. In contrast, early-onset AD (EOAD), diagnosed before age 65, typically arises from autosomal dominant mutations in the genes encoding amyloid precursor protein (APP) or presenilins (PSEN1, PSEN2) [1, 2]. Although LOAD and EOAD share clinical and pathological features, EOAD is often associated with a higher amyloid burden, earlier NFT formation, and faster neurodegeneration [1, 11, 12].

The amyloid hypothesis, first proposed by Hardy and Higgins in 1992 [13], posits that Aβ accumulation initiates AD pathogenesis. This view is supported by several observations: (i) prominent Aβ accumulation in AD brains; (ii) plaque deposition in brain regions associated with learning and memory; (iii) familial AD mutations linked to APP; (iv) the neurotoxicity of Aβ aggregates; and (v) their ability to elicit inflammatory responses [6, 14–17]. Aβ peptides (36–43 amino acids in length) result from sequential cleavage of APP by β-site APP cleaving enzyme 1 (BACE1) and γ-secretase [5]. While Aβ monomers are generally considered non-toxic [18], Aβ oligomers disrupt calcium homeostasis [19], impair synaptic function [20–22], and promote neuronal death [23]. Interestingly, protofibrils and mature fibrils—organized in β-sheet-rich structures—appear less toxic than soluble oligomers, suggesting that fibril formation may buffer against Aβ-induced toxicity [24, 25].

To study AD mechanisms, numerous transgenic animal models expressing mutant human APP—with or without presenilin mutations—have been developed in species ranging from invertebrates to mammals. Commonly used AD mouse models exhibit progressive Aβ accumulation, cognitive deficits, and neuroinflammation, reproducing key features of human AD [26–30]. However, a notable limitation of these models is the early onset of pathology, typically during adolescence or early adulthood, which contrasts with the late-life manifestation seen in humans. Even in familial AD, clinical symptoms rarely appear before the fourth decade of life [31]. This discrepancy raises a fundamental question: does progressive amyloid burden alone drive disease onset, or is the aging brain uniquely susceptible to Aβ toxicity?

Because standard AD models constitutively overexpress mutant APP throughout life, they are poorly suited to investigate how aging modulates Aβ-induced pathology. To address this gap, an alternative model is needed that enables temporal control over APP expression. In the present study, we examined whether the neurological effects of Aβ depend on the age at which mutant APP expression begins. Using a tetracycline-inducible system, we activated expression of a human APP transgene carrying the Swedish and Indiana mutations (APPSweInd) during two distinct adult life stages in mice: mid-age (6–18 months) and old age (12–24 months). After one year of expression, we assessed exploratory behavior, muscle strength, learning ability, amyloid burden, and brain transcriptomes, comparing both groups to age-matched controls with lifelong suppression of APP expression.

Despite similar levels of APP protein, amyloid deposition, and gliosis across age groups, aged APP-expressing mice exhibited more pronounced hyperactivity, cognitive impairment, and muscle weakness compared to their mid-aged counterparts. Transcriptomic analysis revealed a marked downregulation of cholinergic system genes specifically in aged APP mice, confirmed at both RNA and protein levels. Notably, no significant changes were detected in markers of other neuronal cell types, highlighting a selective vulnerability of the cholinergic system in the aging brain.

Together, these findings suggest that age-related susceptibility to Aβ toxicity—rather than amyloid burden alone—drives key aspects of AD pathogenesis, particularly through disruption of cholinergic function. Our inducible APP model provides a valuable platform to dissect how aging renders the brain more vulnerable to Aβ and may help identify therapeutic targets for age-related neurodegeneration.

## Results

### Locomotor hyperactivity, reduced muscle strength, and impairments in spatial and associative learning were more pronounced in APP 12 → 24mo mice compared to APP 6 → 18mo mice

**Fig. 1:**
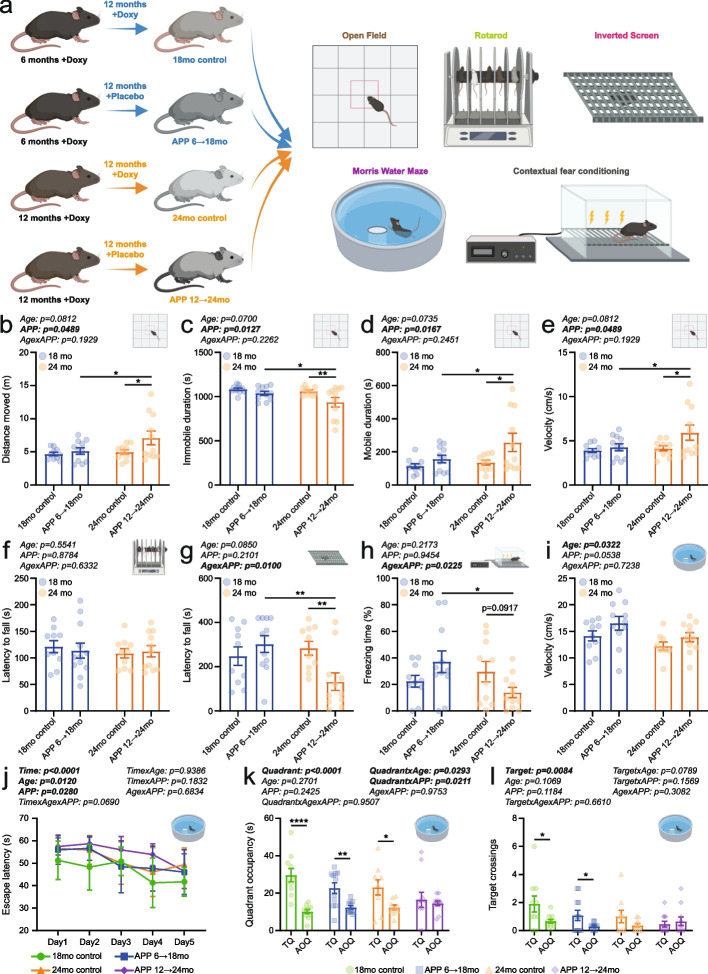
Induction of mutant human APP later in life triggered locomotor hyperactivity, motor deficits, and impairments in associative and spatial learning. **a** Schematic overview of the experimental design. **b** Distance traveled, (**c**) immobile duration, (**d**) mobile duration, and (**e**) movement speed recorded in the open field test (5 male and 5 female 18mo controls: 5 male and 6 female APP 6 → 18mo; 5 male and 5 female 24mo controls; 5 male and 6 female APP 12 → 24mo). **f** Latency to fall on the accelerating rotarod (5 male and 5 female 18mo controls; 6 male and 6 female APP 6 → 18mo; 5 male and 5 female 24mo controls; 5 male and 6 female APP 12 → 24mo). **g** Latency to fall on the inverted screen test (5 male and 5 female 18mo controls; 5 male and 6 female APP 6 → 18mo; 5 male and 5 female 24mo controls; 5 male and 6 female APP 12 → 24mo). **h** Percent time spent freezing during the test session in a contextual fear conditioning paradigm (5 male and 5 female 18mo controls; 5 male and 6 female APP 6 → 18mo; 5 male and 5 female 24mo controls; 5 male and 6 female APP 12 → 24mo). **i** Swim speed and (**j**) escape latency during the training phase of the Morris Water Maze (MWM) (5 male and 5 female 18mo controls; 5 male and 6 female APP 6 → 18mo; 5 male and 4 female 24mo controls; 5 male and 6 female APP 12 → 24mo). (**k)** Time spent in the target quadrant (TQ) vs. the average of all other quadrants (AOQ), and (**l**) number of platform crossings during the MWM probe trial. The panels (**j-l**) were analyzed by three-way ANOVA. Individual data points and group means ± S.E.M. are shown. * *p* < 0.05, ** *p* < 0.01, **** *p* < 0.0001

We aimed to investigate the interaction between mutant APP overexpression and brain aging, under conditions that were not confounded by age-related differences in amyloid burden or by potential developmental effects of early transgene expression. To achieve this, we used a previously established inducible APPSweInd transgenic mouse line [32], based on a Tet-Off system, allowing temporal control of APP expression. APP overexpression was restricted to a one-year window, either from 6 to 18 months of age (APP 6 → 18mo) or from 12 to 24 months (APP 12 → 24mo). Subsequently, behavioral, learning, and memory functions were assessed and compared to age-matched control mice in which mutant APP expression was continuously suppressed via lifelong doxycycline administration (Fig. 1a).

Spontaneous locomotor activity was first assessed using the open field paradigm. Total distance traveled, immobile duration, mobile duration, and velocity were all significantly altered in APP-induced mice, with these effects being more pronounced in APP 12 → 24mo mice compared to APP 6 → 18mo (Fig. 1b–e). In contrast, locomotor activity levels were comparable between 18-month-old and 24-month-old APP-suppressed control mice, indicating that the observed differences were not primarily attributable to age (Fig. 1b–e). Analyses stratified by sex revealed that the APP-induced hyperlocomotion was mainly driven by female APP-overexpressing mice (Supplementary Fig. 1a-d).

Motor coordination and muscle strength were assessed using the accelerating rotarod and the inverted screen test, respectively. Latency to fall on the rotarod did not differ between groups, indicating that motor coordination was unaffected (Fig. 1f; Supplementary Fig. 1e). In contrast, APP 12 → 24mo mice fell from the inverted metal grid significantly earlier than both age-matched controls and APP 6 → 18mo mice, suggesting a specific motor deficit—potentially due to reduced muscle strength—in the APP 12 → 24mo group (Fig. 1g; Supplementary Fig. 1f).

Next, we assessed associative learning and memory using a contextual fear conditioning paradigm in APP 12 → 24mo, APP 6 → 18mo, and age-matched APP-suppressed control mice. On the training day, animals were allowed to explore the conditioning chamber before receiving mild foot shocks delivered through the metal grid floor. The following day, mice were re-exposed to the same chamber to evaluate their conditioned fear responses. Freezing duration was reduced in APP 12 → 24mo mice compared to both APP 6 → 18mo mice and age-matched controls, resulting in a significant interaction between age and APP overexpression (Fig. 1h; Supplementary Fig. 1g).

Finally, spatial learning and memory were assessed using the hidden-platform version of the Morris water maze (Fig. 1i–l; Supplementary Fig. 1h-k). 24-month-old animals swam significantly slower than those in the 18-month-old cohort, while APP-overexpressing mice showed a trend toward increased swim velocity (Fig. 1i). Interestingly, APP-overexpressing females swam faster than age-matched controls, but no difference was found in male animals (Supplementary Fig. 1h). Escape latencies recorded over five days of training revealed significant effects of both age and APP overexpression, with the poorest performance observed in the APP 12 → 24mo group (Fig. 1j; Supplementary Fig. 1i). After the training phase, a probe trial was conducted in which the escape platform was removed. We measured the time each mouse spent in the target quadrant—where the platform had previously been located—and the number of crossings over the former platform location. Analysis of quadrant occupancy showed that APP 6 → 18mo mice spent significantly more time in the target quadrant compared to the average of the other quadrants, similar to the performance of age-matched APP-suppressed controls at both 18 and 24 months (Fig. 1k; Supplementary Fig. 1j). In contrast, APP 12 → 24mo mice exhibited chance-level quadrant occupancy, indicating a failure to retain spatial memory (Fig. 1k; Supplementary Fig. 1j). A similar pattern was observed for target crossing events (Fig. 1l; Supplementary Fig. 1k).

Collectively, our contextual fear conditioning and MWM results indicate that APP transgene expression at different stages of adulthood leads to distinct learning and memory outcomes in mice.

### APP expression levels and cumulative amyloid burden did not differ between APP 6 → 18mo and APP 12 → 24mo mice

**Fig. 2:**
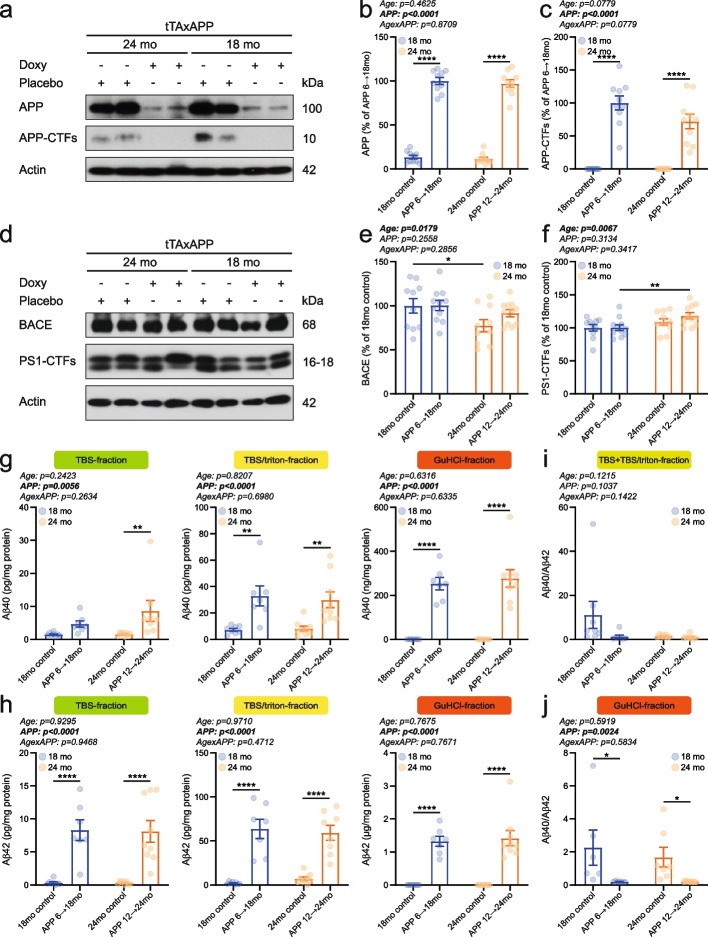
Levels of APP and brain amyloid burden were indistinguishable between APP 6 → 18mo and APP 12 → 24mo mice. **a** Representative western blot results and quantification of (**b**) full-length APP and (**c**) APP C-terminal fragments (APP-CTFs) in 18mo control (5 males and 5 females), APP 6 → 18mo (5 males and 5 females), 24mo control (5 males and 5 females), and APP 12 → 24mo (5 males and 5 females). **d** Representative western blot images and quantification of (**e**) BACE and (**f**) PS1 C-terminal fragments (PS1-CTFs) in 18mo control (5 males and 5 females), APP 6 → 18mo (5 males and 6 females), 24mo control (5 males and 5 females), and APP 12 → 24mo (5 males and 6 females). ELISA-based measurements of (**g**) Aβ40 and (**h**) Aβ42 levels in TBS-fraction (4 male and 4 female 18mo controls; 3 male and 4 female APP 6 → 18mo; 4 male and 4 female 24mo controls; 4–5 male and 4 female APP 12 → 24mo), TBS-triton-fraction (4 male and 4 female 18mo controls; 3 male and 4 female APP 6 → 18mo; 4 male and 5 female 24mo controls; 4–5 male and 4 female APP 12 → 24mo), and GuHCl-fraction (4 male and 4 female 18mo controls; 3 male and 4 female APP 6 → 18mo; 4 male and 5 female 24mo controls; 5 male and 4 female APP 12 → 24mo). **i** Aβ40/Aβ42 ratio calculated for TBS and TBS + Triton X-100 soluble Aβ species. **j** Aβ40/Aβ42 ratio for GuHCl-soluble higher-order Aβ aggregates. Individual data points and group means ± S.E.M. are shown. * *p* < 0.05, ** *p* < 0.01, **** *p* < 0.0001

Following behavioral and cognitive assessments, all animals were sacrificed, and brain tissue was extracted and processed for downstream molecular analyses to validate the model. Consistent with previous reports [32, 33], overexpression of the APPSweInd transgene was effectively suppressed upon doxycycline administration, with minimal leakage (Fig. 2a, b; Supplementary Fig. 2a). Similar levels of full-length APP and C-terminal fragments (CTFs) were detected in animals overexpressing the mutated APP transgene from 6 to 18 months or 12 to 24 months of age (Fig. 2a–c; Supplementary Fig. 2a, b). APP-CTF levels in chronically doxycycline-treated animals were below the detection threshold (Fig. 2c; Supplementary Fig. 2b).

To determine whether APP processing pathways are altered by overexpression of the mutant transgene at different ages, we assessed the expression levels of α-, β-, and γ-secretase components using Western blot and real-time quantitative PCR. Protein abundance of β-secretase (BACE) and PSEN1 C-terminal fragments (PS1-CTFs) was modulated by age but remained independent of APP transgene expression (Fig. 2d–f; Supplementary Fig. 2c, d). Specifically, BACE levels were significantly lower in 24-month-old animals compared to 18-month-old mice, whereas PS1-CTFs showed the opposite trend (Fig. 2d–f; Supplementary Fig. 2c, d). At the transcriptional level, *Adam10* expression was reduced in APP-overexpressing mice, with no significant age-dependent changes (Supplementary Fig. 3a). mRNA levels of β- and γ-secretase components (*Bace1*, *Psen1*, *Psen2*, *Ncstn*, *Psenen*, *Aph1a*, and *Aph1b*) remained largely unaffected by either age or APP transgene expression, with the exception of *Psen2* expression in female mice, which showed an age x APP interaction (Supplementary Fig. 3b–h).

To estimate amyloid burden, we quantified two predominant Aβ species—Aβ40 and Aβ42—in whole brain extracts. Aβ was sequentially extracted using a three-step protocol involving TBS (= soluble), TBS/Triton X-100 (= membrane-associated), and GuHCl (= insoluble) buffers [21]. ELISA-based quantification of Aβ40 and Aβ42 revealed no differences between 18-month-old and 24-month-old APP-overexpressing animals across all three fractions, indicating that 12 months of APP transgene induction resulted in a comparable amyloid load in both APP 6 → 18mo and APP 12 → 24mo mice (Fig. 2g, h; Supplementary Fig. 2e, f). In line with the results above, life-long doxycycline-treated animals exhibited a near-complete absence of amyloid pathology, accumulating only ~ 0.045% of total Aβ40 and Aβ42 compared to age-matched APP-overexpressing mice (Fig. 2g, h; Supplementary Fig. 2e, f). Total brain amyloid burden was 38.48% higher in APP-induced females compared to APP-induced males (Fig. 2g, h; Supplementary Fig. 2e, f). The Aβ40/42 ratio remained unchanged by age in the TBS and TBS/Triton X-100 fractions (Fig. 2i; Supplementary Fig. 2g). However, in the GuHCl fraction, the Aβ40/42 ratio was significantly decreased in APP-induced mice, regardless of age, indicating a marked shift toward Aβ42 biogenesis and deposition independent of the timing of transgene activation (Fig. 2j; Supplementary Fig. 2h).

**Fig. 3:**
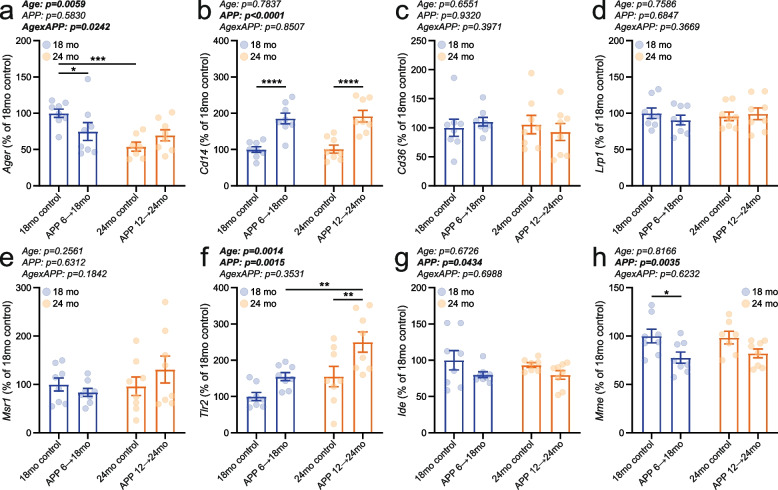
Gene transcription of receptors and enzymes critical for Aβ clearance and degradation was predominantly regulated by APP expression. mRNA levels of (**a**) *Ager*, (**b**) *Cd14*, (**c**) *Cd36*, (**d**) *Lrp1*, (**e**) *Msr1*, (**f**) *Tlr2*, (**g**) *Id*e, and (**h**) *Mme* were measured in the brains of APP 6 → 18mo (4 males and 4 females), APP 12 → 24mo (4 males and 4 females), and age-matched control animals (4 male and 4 female 18mo controls; 4 male and 3–4 female 24mo controls).Individual data points and group means ± S.E.M. are shown. * *p* < 0.05, ** *p* < 0.01, *** *p* < 0.001, **** *p* < 0.0001

In addition to Aβ biogenesis and deposition, we investigated whether receptors and enzymes involved in Aβ clearance and degradation were modulated by age and/or APP transgene overexpression. Our qPCR-based analyses revealed that mRNA levels of several Aβ-binding receptors were altered by both age and APP transgene expression (Fig. 3a–f; Supplementary Fig. 4a-f). Transcriptional levels of *Ager* were reduced in 24-month-old animals, independent of APP induction status (Fig. 3a; Supplementary Fig. 4a). *Cd14* expression was increased upon APP overexpression, regardless of age and sex (Fig. 3b; Supplementary Fig. 4b). In contrast, expression of *Cd36* and *Lrp1* remained unchanged (Fig. 3c, d; Supplementary Fig. 4c, d). Opposing aging-associated alterations in *Msr1* transcription were detected in a sex-specific manner (Fig. 3e; Supplementary Fig. 4e). Less *Msr1* mRNA was present in older male mice, whereas higher *Msr1* expression was found in older female animals (Supplementary Fig. 4e). *Tlr2* expression was influenced by both age and APP overexpression, with age-related increases further amplified in APP-overexpressing mice irrespective of sex (Fig. 3f; Supplementary Fig. 4f). Additional quantification of *Ide* and *Mme*, which encode insulin-degrading enzyme (IDE) and neprilysin (NEP), respectively, showed significantly lower transcript levels in APP-overexpressing mice compared to controls, with no apparent effect of age (Fig. 3g, h). Stratification by sex indicated that the APP-induced decrease in *Ide* and *Mme* expression was more pronounced in males than in females (Supplementary Fig. 4g, h).

Taken together, one year of APPSweInd transgene induction—restricted to either 6–18 months or 12–24 months of age—resulted in comparable levels of full-length APP, APP-CTFs, and brain amyloid deposits. The abundance of secretase components and key elements involved in Aβ clearance and degradation was often influenced by age, while some targets were similarly up- or downregulated in response to APP transgene induction in both APP 6 → 18mo and APP 12 → 24mo animals. Thus, the mouse cohorts we generated represent a valid model for investigating how age modulates neurological and behavioral phenotypes driven by mutant APP overexpression, while controlling for both the duration of transgene activation and cumulative amyloid burden across age groups.

### The effects of APP transgene induction on inflammatory cytokines and chemokines were similar in APP 6 → 18mo and APP 12 → 24mo mice

**Fig. 4:**
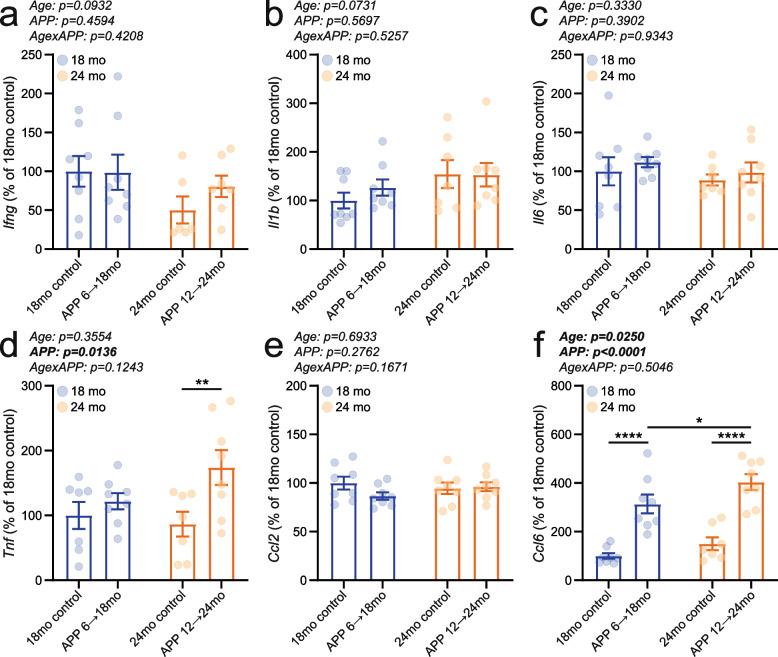
Inflammatory cytokine and chemokine transcription was enhanced in response to mutant APP expression. mRNA levels of (**a**) *Ifng*, (**b**) *Il1b*, (**c**) *Il6*, (**d**) *Tnf*, (**e**) *Ccl2*, and (**f**) *Ccl6* were measured in the brains of APP 6 → 18mo (4 males and 4 females), APP 12 → 24mo (3–4 males and 4 females), and age-matched control animals (3–4 male and 4 female 18mo controls; 4 male and 2–4 female 24mo controls). Individual data points and group means ± S.E.M. are shown. * *p* < 0.05, ** *p* < 0.01, **** *p* < 0.0001

Given that persistent immune activity is a key pathological feature of Alzheimer's disease [17], we measured mRNA levels of selected cytokines and chemokines with established roles in inflammation. An age x APP interaction in *Ifng* expression was observed only in male mice, but not in females (Fig. 4a; Supplementary Fig. 5a). *Il1b* transcript levels showed a trend toward increase in 24-month-old animals, but this did not reach statistical significance (Fig. 4b; age: p = 0.0731; Supplementary Fig. 5b). Gene transcription of *Il6* and *Ccl2* did not differ between groups (Fig. 4c, e; Supplementary Fig. 5c, e). *Tnf* expression was significantly upregulated in APP-induced mice (Fig. 4d; Supplementary Fig. 5d). Both age and APP transgene induction elevated *Ccl6* transcription, with a stronger effect observed for APP induction than for aging (Fig. 4f; Supplementary Fig. 5f).

### Autophagic activity is differentially regulated by aging and APP overexpression

Autophagy plays a dual role in Alzheimer’s disease, contributing to both Aβ release and clearance [34]. Intact autophagic function is essential for the effective sequestration of Aβ, thereby preventing abnormal accumulation of this toxic peptide within neurons [35]. To investigate this, we analyzed key proteins involved in autophagosome formation by Western blot.

**Fig. 5:**
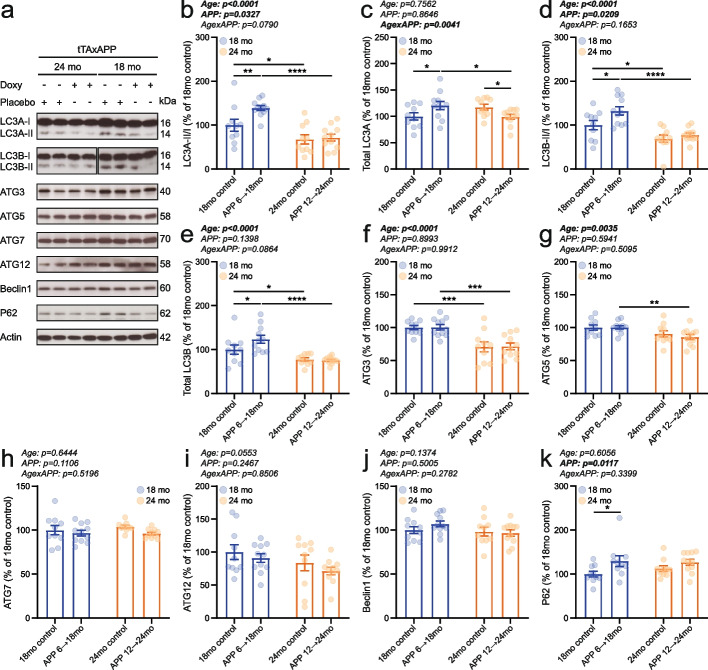
Autophagic activity in the mouse brain was modulated by both aging and mutant APP transgene expression. **a** Representative western blot images are shown. Autophagy-related targets assessed include (**b**) LC3A-II/LC3A-I ratio, (**c**) total LC3A, (**d**) LC3B-II/LC3B-I ratio, (**e**) total LC3B, (**f**) ATG3, (**g**) ATG5, (**h**) ATG7, (**i**) ATG12, (**j**) Beclin-1, and (**k**) SQSTM1/p62. Sample size was 5 male and 5 female 18mo controls, 4–5 male and 6 female APP 6 → 18mo, 4–5 male and 5 female 24mo controls, and 5 male and 5–6 female APP 12 → 24mo. Individual data points and group means ± S.E.M. are presented. * *p* < 0.05, ** *p* < 0.01, *** *p* < 0.001, **** *p* < 0.0001

Both LC3A-II/I and LC3B-II/I ratios were lower in 24-month-old mice compared to 18-month-old individuals, consistent with reduced autophagic activity at older age (Fig. 5a, b, d; Supplementary Fig. 6a, c). Interestingly, these ratios were elevated in 18-month-old APP-induced mice relative to age-matched APP-suppressed controls, whereas no measurable difference was observed between the 24-month-old groups (Fig. 5a, b, d; Supplementary Fig. 6a, c). Total LC3A levels showed a significant age × APP interaction and total LC3B exhibited an age-related decrease (Fig. 5a, c, e; Supplementary Fig. 6b, d). Downregulation of ATG3 and ATG5 was observed at 24 months of age, independent of APP overexpression (Fig. 5f, g; Supplementary Fig. 6e, f). Lower abundance of ATG7 was present in the male APP-induced mice, but not in female APP-overexpressing animals (Fig. 5h; Supplementary Fig. 6g). Protein levels of ATG12 and Beclin-1 were not significantly affected by either age or APP transgene induction (Fig. 5i, j; Supplementary Fig. 6h, i). To assess autophagic flux, we measured SQSTM1/p62 levels as an indicator of turnover efficiency. SQSTM1/p62 abundance was significantly increased in APP-induced mice, with no apparent effect of age, suggesting impaired autophagic degradation associated with APP overexpression (Fig. 5k; Supplementary Fig. 6j).

### Molecular analyses identified the brain’s cholinergic system as selectively vulnerable to mutant APP induction at advanced age

**Fig. 6:**
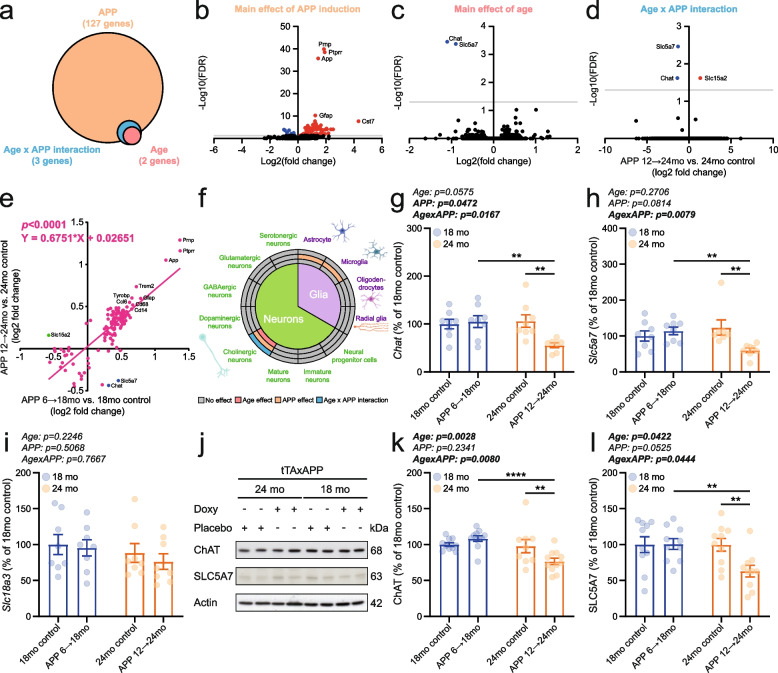
Selective vulnerability of the aged mouse cholinergic system to induced mutant APP expression. **a** Whole-brain RNA sequencing identified genes with a main effect of mutant APP expression, a main effect of age, and/or a significant age × APP interaction (FDR < 0.05). Samples used for differential gene expression analyses included 2 male and 2 female 18mo controls, 2 male and 2 female APP 6 → 18mo, 2 male and 1 female 24mo controls, and 2 male and 2 female APP 12 → 24mo. Volcano plots showing differentially expressed genes driven by (**b**) mutant APP expression, (**c**) age, and (**d**) an age × APP interaction in the 24-month-old mice (APP 12 → 24mo vs. 24-month-old control). **e** Correlation analysis of APP effect sizes on gene expression between 24-month-old and 18-month-old animals. **f** Overview of age and APP-related changes in brain cell lineage marker genes. mRNA levels of (**g**) *Chat*, (**h**) *Slc5a7*, and (**i**) *Slc18a3* were measured by qPCR in 18mo control (4 males and 4 females), APP 6 → 18mo (4 males and 4 females), 24mo control (4 males and 3–4 females), and APP 12 → 24mo (4 males and 3–4 females). **j** Representative western blot images of cholinergic marker proteins. Protein levels of (**k**) ChAT and (**l**) SLC5A7 were specifically reduced in APP-induced 24-month-old mice. Sample size corresponds to 5 male and 5 female 18mo controls, 5 male and 5–6 female APP 6 → 18mo, 4–5 male and 5 female 24mo controls, and 4–5 male and 6 female APP 12 → 24mo. Individual data points and group means ± S.E.M. are presented. ** *p* < 0.01, **** *p* < 0.0001

To investigate the molecular mechanisms underlying the interaction between brain aging and mutant human APP overexpression at the behavioral and cognitive levels, we performed RNA-seq-based transcriptomic analyses. This unbiased approach aimed to identify gene expression changes driven by the combined effects of aging and APP overexpression. Applying a false discovery rate (FDR) threshold of 0.05, we identified 127 differentially expressed genes (DEGs), including 124 protein-coding and 3 non-coding transcripts, associated with APP overexpression. Additionally, 2 genes showed a main effect of age, and 3 genes exhibited a significant interaction between age and APP expression (Fig. 6a; Supplementary Data 1).

The top 10 DEGs affected by APP overexpression included *App*, *Ccl6*, *Cd14*, *Cd68*, *Cst7*, *Gfap*, *Prnp*, *Ptprr*, *Trem2*, and *Tyrobp* (Fig. 6b). Notably, 77 out of the 124 protein-coding DEGs (62.1%) regulated by APP overexpression are also annotated in transcriptomic datasets from human AD patients (Supplementary Table 1) [36]. Functional analysis of canonical pathways, diseases and biological functions, and upstream regulators using Ingenuity Pathway Analysis (IPA) confirmed that these transcriptional changes are enriched in inflammatory and immune activation processes (Supplementary Fig. 7; Supplementary Data 2).

Two DEGs—*Chat* (encoding choline acetyltransferase, ChAT) and *Slc5a7* (encoding the choline transporter SLC5A7, also known as the high-affinity choline transporter 1, CHT1)—exhibited both a main effect of age and a significant age x mutant APP interaction (Fig. 6c, d; Supplementary Data 1). For both genes, expression levels were selectively reduced in APP 12 → 24mo mice. Another gene, *Slc15a2* (encoding a proton-coupled peptide transporter), also showed a significant age x APP interaction; however, in contrast to *Chat* and *Slc5a7*, its expression trajectory was reversed in older mice with mutant APP induction (Fig. 6d; Supplementary Data 1). Correlation analysis of all DEGs revealed a general similarity in gene expression changes driven by APP overexpression across age groups, whereas the expression patterns of *Chat*, *Slc5a7*, and *Slc15a2* were distinctly different between APP 12 → 24mo and APP 6 → 18mo mice (Fig. 6e).

Next, we examined our RNA sequencing dataset to determine whether gene expression profiles of specific brain cell lineages were affected by aging and/or mutant APP expression. Among glial cells, analysis of lineage-specific markers revealed a significant main effect of mutant APP on astrocytes and microglia, whereas oligodendrocytes and radial glia were unaffected (Fig. 6f; Supplementary Table 2). Upregulation of astrocyte (*Gfap*) and microglia (*Itgam*, *Trem2*, and *Cd68*) lineage markers following mutant APP induction was further validated by qPCR, showing consistent results irrespective of age and sex (Supplementary Fig. 8). Additional evidence of APP-induced gliosis was provided by quantifying GFAP, CD11b, CD68, IBA1, and TREM2 by western blot. APP-overexpression between 6 → 18 months or 12 → 24mo led to a comparable elevation of these proteins in both male and female mice (Supplementary Fig. 9).

In contrast, among neuron-specific lineage markers, only cholinergic neurons exhibited significant changes related to age or mutant APP expression (Fig. 6f; Supplementary Table 2). To validate the RNA-seq findings, we quantified mRNA levels of three cholinergic neuron markers—*Chat*, *Slc5a7*, and *Slc18a3* (encoding the vesicular acetylcholine transporter, VAChT)—using qPCR. Gene expression levels of *Chat* and *Slc5a7* were specifically reduced in APP 12 → 24mo mice, with no significant differences observed in other groups (Fig. 6g, h; Supplementary Fig. 10a, b). In contrast, *Slc18a3* mRNA levels remained unchanged, consistent with the RNA sequencing data (Fig. 6i; Supplementary Fig. 10c). Additional evaluation of ChAT and SLC5A7 at the protein level confirmed their specific reduction in the APP 12 → 24mo group (Fig. 6j–l; Supplementary Fig. 10d, e). In contrast, levels of general synaptic marker proteins (PSD95 and synaptophysin) were decreased in the 24-month-old groups but showed no measurable effect of mutant APP expression (Supplementary Fig. 11).

## Discussion

Alzheimer’s disease typically manifests in late life [10], and brain amyloid pathology—accumulating progressively during the aging process [37]—has been proposed to play a key pathogenic role. However, it remains unresolved whether aging increases Alzheimer’s disease risk primarily by enabling the time-dependent buildup of amyloid, or by rendering neural tissues more susceptible to amyloid toxicity. In this study, we addressed this question using an inducible mutant APP mouse model, which allowed us to restrict APP expression to defined life stages (either from 6 to 18 months or from 12 to 24 months). We then assessed behavioral, cognitive, and molecular outcomes, including APP processing, brain amyloid burden, Aβ clearance, inflammation, autophagy, and whole-brain transcriptomic changes.

Our findings demonstrate that mutant APP-related behavioral and cognitive impairments—including hyperlocomotion, motor deficits, and learning and memory dysfunction—were more pronounced in the APP 12 → 24mo group compared to the APP 6 → 18mo mice. Subsequent molecular analyses revealed that these differences were not attributable to variations in brain amyloid burden, Aβ clearance, inflammatory responses, or autophagic activity. Instead, APP overexpression in late life selectively disrupted key components of the brain’s cholinergic system, whereas these targets remained unaffected in the earlier APP induction group. Notably, markers of brain gliosis were elevated following APP induction in both age groups. Collectively, our data indicate that the timing of human mutant APP overexpression critically shapes disease progression, with age-dependent disruption of the central cholinergic system emerging as a key feature of increased vulnerability.

To model disease progression in humans and disentangle the complex relationship between cumulative amyloid deposition and brain aging in Alzheimer’s disease, we induced APP expression either during midlife (from 6 to 18 months of age) or in late life (from 12 to 24 months of age). Early-life induction of mutant APP was deliberately avoided to eliminate potential neurodevelopmental effects from confounding the analysis. As shown in our previous work, the vast majority of aging-associated alterations emerge during the second half of the mouse lifespan, with age-related changes in the brain becoming largely detectable at 20 months of age or later [38, 39]. Accordingly, overexpressing APP from 12 to 24 months substantially overlaps with the period when brain aging becomes evident, whereas APP induction from 6 to 18 months occurs during a phase less influenced by aging-related changes in the brain.

As expected, expression of the mutant APP transgene was effectively suppressed by continuous doxycycline administration (Fig. 2a), consistent with previous reports [33, 40]. We also confirmed that the abundance of full-length APP, levels of APP C-terminal fragments (APP-CTFs), and total brain amyloid burden were independent of the age at onset in our experimental setup (Fig. 2b, c, g, h). ELISA-based quantification of the predominant Aβ species—Aβ40 and Aβ42—revealed an overrepresentation of Aβ42 (Fig. 2g, h), a pattern typically observed in AD mouse models expressing mutant human APP [41–44]. Moreover, amyloid peptides were predominantly deposited as insoluble, higher-order aggregates (Fig. 2g, h), in line with findings from other AD mouse lines [41–44]. Thus, the amyloid pathology observed in our model closely resembles the disease characteristics found in constitutively APP-overexpressing AD mouse models. The greater brain amyloid burden in females was associated with overall more pronounced hyperactivity, consistent with sex-specific locomotor impairments arising from differential Aβ load.

Progressive amyloid deposition in AD is driven by an imbalance between Aβ production and clearance during disease progression, largely due to the declining efficiency of the Aβ clearance machinery in old age [13, 45]. To investigate the influence of age and mutant APP expression on the Aβ clearance machinery in our model, we measured gene expression levels of six receptors known to mediate Aβ uptake: *Ager*, *Cd14*, *Cd36*, *Lrp1*, *Msr1*, and *Tlr2* [46–52]. Increased expression of the receptor for advanced glycation end products (RAGE), encoded by *AGER*, has been reported in AD patients [53]. In contrast, our AD mouse model revealed an age-related decrease in *Ager* expression, with minimal differences between 24-month-old APP-induced and APP-suppressed animals (Fig. 3a). Transcriptional activity of *Cd36*, *Lrp1*, and *Msr1* remained unchanged across age groups and APP expression status (Fig. 3c–e). In contrast, *Cd14* and *Tlr2* expression were significantly upregulated in APP-expressing animals (Fig. 3b, f), consistent with findings from other AD mouse models [47, 51, 54]. We also assessed *Ide* and *Mme*, two genes encoding key Aβ-degrading enzymes [55, 56]. mRNA levels of both *Ide* and *Mme* were significantly reduced in APP-induced animals (Fig. 3g, h), aligning with observations from post-mortem human AD brains [55]. Importantly, total amyloid burden and Aβ species levels were equivalent between the two APP-induced groups, indicating that differences in behavioral and cognitive outcomes are not explained by differences in amyloid accumulation.

Chronic inflammation is a well-established hallmark of both aging and AD [6, 16, 57, 58]. Microglia are key contributors to neuroinflammation in AD, and preclinical studies have shown that inhibiting microglial activation significantly alleviates AD-related symptoms—highlighting inflammation as a promising therapeutic target [6, 17, 42, 44, 58]. Although increased inflammation is a common feature across many AD mouse models, the temporal dynamics, molecular targets, and magnitude of activation can vary substantially [42, 59–61]. In the present study, gene transcription of *Tnf* and *Ccl6* was upregulated in APP-induced mouse brains, while the remaining four cytokines and chemokines assessed did not show significant changes (Fig. 4). RNA sequencing and subsequent analyses further confirmed elevated neuroinflammation in APP-expressing animals, independent of age (Fig. 6e; Supplementary Fig. 5; Supplementary Data 1), suggesting that age-dependent variations in the neuroinflammatory response to mutant APP expression are unlikely to account for the behavioral differences observed across age groups.

Autophagy is the primary pathway for recycling excessive or dysfunctional cellular components via lysosomal degradation—a vital adaptive response that enables cells to cope with stress and nutrient deprivation [62–65]. Macroautophagy, in principle, can be divided into several phases: the initial formation of a phagophore (also known as nucleation), the engulfment of cargo through membrane elongation, and the subsequent fusion of the autophagosome with a lysosome to form an autolysosome (also known as an autophagolysosome), where degradation of the sequestered material occurs [62, 66]. Numerous studies across species have identified impaired autophagy as a hallmark of aging [57, 67–70]. Consequently, enhancing autophagy has emerged as a potent intervention strategy to extend lifespan in model organisms such as worms, flies, and mice [66, 71, 72].

Autophagy in the context of AD presents a paradox: while Aβ stimulates autophagosome formation, these vesicles accumulate as autophagy intermediates (autophagic vacuoles) within neurites, reflecting a failure in autophagic flux [73, 74]. Mechanistically, this disruption has been linked to defective acidification of autolysosomes, which promotes intraneuronal Aβ accumulation and contributes to senile plaque formation [75]. To investigate whether altered autophagy is associated with the behavioral and cognitive differences observed in our model, we analyzed several autophagy-related marker proteins. The LC3-II/I ratio, total LC3 abundance, and protein levels of ATG3 and ATG5 were generally reduced in the 24-month-old groups (Fig. 5a–g), indicating diminished autophagic activity with age, independent of APP expression. Interestingly, 18-month-old APP-induced mice showed an increased LC3-II/I ratio compared to age-matched APP-suppressed animals, whereas no differences were detected in the 24-month-old groups (Fig. 5a, b, d). A similar increase in LC3-II/I ratio has previously been reported in APP/PS1 mice [76]. Additionally, levels of p62/SQSTM1—a marker of autophagic efficiency [77]—were elevated in APP-induced groups (Fig. 5k), supporting the presence of an age-independent autophagic blockade in response to Aβ in our mouse model. These findings indicate that while aging impairs autophagosome formation and APP overexpression disrupts autophagic degradation, the resulting impairment in autophagic flux is comparable across APP-induced groups—suggesting that autophagy dysfunction alone is unlikely to account for the age-dependent behavioral differences observed in our model.

After finding no evidence that brain amyloid burden, inflammation, or autophagy accounted for the behavioral and cognitive differences observed between 18-month-old and 24-month-old APP-induced and APP-suppressed mice, we performed unbiased whole-brain RNA sequencing to uncover alternative mechanistic explanations. Genes differentially regulated by the APP transgene revealed a transcriptional profile dominated by inflammatory activation and gliosis, both of which are well-established features of AD pathology (Fig. 6b, f; Supplementary Fig. 7). Subsequent qPCR- and western blot based analyses of astrocytic and microglial lineage markers confirmed that APP-induced gliosis occurred independently of age and sex (Supplementary Fig. 8, 9). Notably, only three genes were differentially expressed due to age or showed a significant age × APP interaction (Fig. 6a); two of these—*Chat* and *Slc5a7*—are markers of cholinergic neurons (Fig. 6c, d, f). The third gene, *Slc15a2*, encodes the proton-coupled oligopeptide transporter PEPT2, whose role in AD remains unexplored. The relative low number of DEGs associated with age or displaying a significant age × APP interaction is likely explained by our experimental framework in which old vs. very old animals were compared. We validated the specific downregulation of *Chat* and *Slc5a7* in APP 12 → 24mo mice using independent analyses at both the transcript and protein levels (Fig. 6j–l), highlighting the selective vulnerability of the cholinergic system to APP overexpression in the aging brain.

Among all neuronal subpopulations, cholinergic neurons—particularly those located in the basal forebrain—are known to be exceptionally vulnerable in the brains of AD patients and in AD animal models. In patients with AD, a marked decline in choline acetyltransferase (ChAT) and acetylcholinesterase (AChE) activity typically precedes the substantial loss of cholinergic neurons in the basal forebrain as the disease progresses [9, 78]. The degeneration of cholinergic neurons correlates closely with the progression of cognitive decline in AD, as well as in other human neurodegenerative disorders [79]. However, AD mouse models expressing mutant human APP isoforms do not fully replicate this aspect of human pathology. As reported in previous studies, the number of cholinergic neurons in these models remains largely stable throughout life, while changes in neuronal volume emerge during early to mid-life stages, and ChAT activity declines only at advanced age [80–83]. The molecular mechanisms underlying the selective vulnerability of cholinergic neurons in AD remain incompletely understood. Proposed mechanisms include the intraneuronal accumulation of Aβ oligomers [84], Aβ-induced activation of apoptosis via interaction with the p75 neurotrophin receptor [85], and dysregulation of neurotrophic signaling and transport [86]. These pathogenic processes are believed to contribute to the progressive denervation of cholinergic terminals in the hippocampus and cortex [87].

In sum, our findings demonstrate that the cholinergic system in the mouse brain is selectively impaired following a one-year induction of the APPSweInd transgene beginning at 12 months of age, whereas no such impairment was observed when transgene expression was initiated at 6 months. The underlying mechanisms may involve downregulation of cholinergic markers or a potential loss of cholinergic neurons in 24-month-old APP-induced mice. However, a functional decline—rather than widespread neuronal loss—appears more likely, given that changes were limited to a subset of cholinergic markers (*Chat* and *Slc5a7*), while others (*Ache* and *Slc18a3*) remained unchanged. This study provides new insights into the complex interplay between brain aging and cumulative amyloid deposition in driving AD progression. Our findings offer a valuable foundation for future preclinical and clinical investigations into age-related vulnerability in AD.

## Materials and methods

### Ethical statement

This study was approved by the Landesamt für Natur, Umwelt und Verbraucherschutz Nordrhein-Westfalen (Recklinghausen, Germany) (in accordance with the German Animal Welfare Act) and the Chancellor’s Animal Research Committee at Qingdao University (in accordance with National Institutes of Health guidelines).

### Animals

Male mice overexpressing the tetO-APPswe/ind transgene (B6.Cg-Tg(tetO-APPSweInd)102Dbo/Mmjax; stock no. 34845-JAX; Jackson Laboratory, Bar Harbor, MA, USA) on a C57BL/6 background were obtained from Jackson Laboratory. These males were bred with females carrying a tetracycline transactivator under the control of the calcium/calmodulin-dependent protein kinase II alpha (Camk2α-tTA) promoter (B6.Cg-Tg(Camk2a-tTA)1Mmay/DboJ; stock no. 007004; Jackson Laboratory, Bar Harbor, MA, USA) to generate the double-transgenic offspring used in this study.

Animals were group-housed in individually ventilated cages and maintained under specific pathogen-free conditions. They were kept at a constant temperature of 22 °C, under a 12 h:12 h light/dark cycle, with continuous access to food and water. All procedures complied with local and federal animal welfare regulations.

### Doxycycline treatment

Doxycycline (Doxy) was administered by supplementing the chow with 200 mg/kg doxycycline (SM R/M-H diet, 10 mm pellets; Ssniff, Soest, Germany). Breeding pairs—tetO-APPswe/ind males and Camk2α-tTA females—were maintained on Doxy-supplemented chow to suppress APP transgene expression during embryonic development and lactation. After weaning, all offspring continued to receive Doxy-containing chow until APP transgene expression was induced by switching to the corresponding control diet lacking Doxy.

### Experimental design

Two cohorts of double-transgenic mice were generated. In the first cohort, animals were maintained on doxycycline (Doxy)-supplemented chow until 12 months of age. At that point, half of the mice were switched to control chow for an additional 12 months to induce APP transgene expression (24-month-old APP group), while the other half remained on Doxy-supplemented chow to maintain transgene suppression (24-month-old control group).

In parallel, the second cohort was raised on Doxy-supplemented chow until 6 months of age. Subsequently, half of the animals were switched to control chow and maintained on it until 18 months of age (18-month-old APP group), while the remaining animals continued on Doxy chow throughout (18-month-old control group).

Behavioral testing began at 21 months for the 24-month cohort and at 15 months for the 18-month cohort. All groups were tested in parallel. Behavioral analyses were initiated with the following group sizes, using approximately balanced sex ratios in all groups: APP 12 → 24mo, *n* = 11 mice; APP 6 → 18mo, *n* = 12 mice; 18mo control, *n* = 10 mice; 24mo control, n = 10 mice. Following behavioral assessments, animals were sacrificed at 24 or 18 months of age, respectively.

### Open field

Locomotor and exploratory activity of the mice was assessed using an open field test, as previously described with minor modifications [88–90]. Briefly, each animal was placed in an individual acrylic box and allowed to explore the arena for 20 min. Lateral movements were recorded using an automated video tracking system (EthoVision XT, Noldus, Wageningen, Netherlands). Parameters analyzed included total distance traveled, duration of mobility, duration of immobility, and average velocity.

### Rotarod

Motor coordination was assessed using an accelerating rotarod apparatus (Med Associates, Fairfax, VT, USA) as previously described [91–93]. Mice were placed on a rotating beam that continuously accelerated from 4 to 40 rpm. Each trial ended when the mouse either fell off the beam, displayed clear signs of passive cycling (i.e., clinging to the beam without active movement), or reached a maximum duration of 5 min, whichever occurred first. Animals underwent three trials per day over three consecutive days, and the mean latency to fall was calculated as the average across all trials.

### Inverted screen test

Muscle strength was assessed using the inverted screen test. Mice were placed on a metal grid, which they grasped with all four limbs. The grid was then inverted, suspending the animals approximately 30 cm above their home cage. The latency to fall was recorded for each trial. Testing was conducted over three consecutive days, with each animal undergoing three trials per day. Each trial had a maximum duration of 7 min. We report mean latencies to fall, averaged across all sessions.

### Morris water maze

Spatial learning ability was assessed using the hidden-platform version of the Morris water maze (MWM), as previously described [94–96]. Each mouse underwent six training trials per day, starting from different positions, over a period of five consecutive days. Trials ended when the mouse climbed onto the escape platform (10 cm in diameter; located approximately 0.7 cm below the water surface) and remained there for at least one second, or when 60 s had elapsed. Escape latencies were recorded during training using an automated tracking system (EthoVision XT, Noldus, Wageningen, Netherlands).

Following the training phase, a 1-min probe trial was conducted to assess memory retention of the platform’s location. During this trial, the escape platform was removed, and mice were released from a start point located in the quadrant opposite to the former platform location. The time spent in the target quadrant—where the platform had previously been located—was measured and compared to the time spent in the other quadrants. In addition, the number of crossings over the former platform location and swim speed were recorded.

### Contextual fear conditioning

Contextual fear conditioning—a widely used test to assess associative learning deficits—was performed using a near-infrared video tracking system (Med Associates, Fairfax, VT, USA), as previously described [95, 97]. The training session lasted 184 s and included two mild foot shocks (0.75 mA, 2 s duration), administered at 60 and 120 s via a metal grid on the chamber floor. On the following day, mice were re-exposed to the same context for an identical duration, but without receiving any shocks. Prolonged immobility (freezing) during the test session was interpreted as an indicator of successful associative learning.

### Brain tissue preparation

Mice were sacrificed by cervical dislocation. Brain hemispheres were dissected, snap-frozen in liquid nitrogen, and stored at –80 °C until further use. For downstream processing, one frozen hemisphere was pulverized in liquid nitrogen using a porcelain mortar and pestle set (MTC Haldenwanger, Waldkraiburg, Germany). All equipment was pre-cooled and kept on dry ice throughout the procedure to maintain consistent low temperatures. The resulting brain tissue powder was promptly transferred into pre-chilled tubes and stored at –80 °C.

### Aβ ELISA

Extraction of Aβ was performed following a previously described protocol with minor modifications [59]. Briefly, frozen brain tissue powder was homogenized in Tris-buffered saline (TBS, pH 7.4) containing 1 × protease inhibitor cocktail and 1 × phosphatase inhibitor cocktail (both from Roche Applied Bioscience, Germany). After centrifugation at 15,000 × g for 30 min at 4 °C, the supernatant (TBS fraction) was collected and stored at –80 °C. Next, the remaining pellet was resuspended in TBS containing 1% Triton X-100 (Sigma-Aldrich, Taufkirchen, Germany), along with protease and phosphatase inhibitors. The suspension was incubated on ice for 30 min with occasional mixing. After a second centrifugation under the same conditions, the resulting supernatant (TBS/Triton fraction) was collected and stored at –80 °C. To extract Aβ from the TBS/Triton-insoluble pellet, an ice-cold guanidine hydrochloride (GuHCl) solution (5 M GuHCl + 50 mM Tris, pH 8.0; Sigma-Aldrich) was added, and the mixture was incubated overnight at 25 °C with shaking at 700 rpm. The resulting turbid solution (GuHCl fraction) was stored at –80 °C until further use. Protein concentrations in the TBS, TBS/Triton, and GuHCl fractions were determined using the Pierce BCA Protein Assay Kit (Thermo Fisher Scientific, Dreieich, Germany). The levels of Aβ40 and Aβ42 in each fraction were quantified using human-specific ELISA kits (Thermo Fisher Scientific), according to the manufacturer’s instructions. Aβ concentrations were normalized to the total protein content of each respective sample.

### Immunoblotting

Immunoblotting was performed as previously described [38, 98, 99]. 20 µg of protein was loaded onto self-cast Tris–glycine sodium dodecyl sulfate (SDS) gels and separated by electrophoresis, followed by transfer onto nitrocellulose membranes with a 0.1 µm pore size (GE Healthcare, Little Chalfont, UK). Membranes were blocked for 1 h at room temperature in blocking buffer consisting of phosphate-buffered saline (PBS) containing 10% skim milk (Carl Roth, Karlsruhe, Germany) to reduce non-specific binding. After brief rinses in PBS, membranes were incubated overnight at 4 °C with primary antibodies. Following multiple PBS washes, secondary antibodies were applied for 1 h at room temperature. After final washes, immune-reactive signals were visualized using enhanced chemiluminescence (Amersham ECL Western Blotting Detection Reagents; GE Healthcare) and Amersham Hyperfilm ECL (GE Healthcare). Densitometric analysis was performed using ImageJ software (version 1.52i). Target protein levels were normalized to actin detected in the same lane.

The following primary antibodies were used: mouse monoclonal anti-human Aβ (#SIG-39300, clone 6E10, 1:2,000; Covance, Princeton, NJ, USA), rabbit monoclonal anti-BACE1 (#5606, clone D10E5, 1:2,000; Cell Signaling Technology, Danvers, MA, USA), rabbit monoclonal anti-presenilin 1 (#5643, clone D39D1, 1:3,000; Cell Signaling Technology), rabbit monoclonal anti-LC3A (#4599, clone D50G8, 1:2,000; Cell Signaling Technology), rabbit polyclonal anti-LC3B (#2775, 1:2,000; Cell Signaling Technology), rabbit polyclonal anti-ATG3 (#3415, 1:2,000; Cell Signaling Technology), rabbit monoclonal anti-ATG5 (#8540, clone D1G9, 1:2,000; Cell Signaling Technology), rabbit monoclonal anti-ATG7 (#8558, clone D12B11, 1:2,000; Cell Signaling Technology), rabbit monoclonal anti-ATG12 (#4180, clone D88H11, 1:2,000; Cell Signaling Technology), rabbit monoclonal anti-beclin 1 (#3495, clone D40C5, 1:2,000; Cell Signaling Technology), rabbit polyclonal anti-p62 (#5114, 1:1,500; Cell Signaling Technology), rabbit monoclonal anti-ChAT (#ab181023, clone EPR13024(B), 1:2,000; Abcam, Cambridge, UK), rabbit polyclonal anti-SLC5A7 (#ab135043, 1:3,000; Abcam), rabbit polyclonal anti-PSD95 (#2507, 1:2,000; Cell Signaling Technology), mouse monoclonal anti-synaptophysin (#ab8049, clone SY38, 1:2,000; Abcam), rabbit anti GFAP (#12389, clone D1F4Q, 1:2,000; Cell Signaling Technology), rabbit anti CD11b (#17800, clone E6E1M, 1:2,000; Cell Signaling Technology), rabbit anti CD68 (#97778, clone E3O7V, 1:2,000; Cell Signaling Technology), rabbit anti Iba1/AIF-1 (#17198, clone E4O4W, 1:2,000; Cell Signaling Technology), rabbit anti TREM2 (#59621, clone E9O9F, 1:2,000; Cell Signaling Technology), mouse monoclonal anti-actin (#869100, clone C4, 1:20,000; MP Biomedicals, Santa Ana, CA, USA). The following secondary antibodies were used: horseradish peroxidase (HRP)-conjugated goat anti-mouse (1:3,000; Agilent, Santa Clara, CA, USA), HRP-conjugated goat anti-rabbit (1:3,000; Promega, Madison, WI, USA).

### RNA extraction

RNA was isolated using a two-step protocol combining peqGold TriFast (Peqlab, Erlangen, Germany) and the RNeasy Mini Kit (Qiagen, Hilden, Germany). Frozen brain tissue powder was homogenized in 1 ml peqGold TriFast solution and kept on ice until all samples were processed. Samples were then incubated at room temperature for 5 min, followed by centrifugation at 12,000 × g for 10 min at 4 °C. The supernatant was transferred to a new tube, and 200 µl chloroform (Sigma-Aldrich, Taufkirchen, Germany) was added. Samples were vigorously shaken and incubated for another 5 min at room temperature. After repeating the centrifugation step, the upper aqueous phase—containing the RNA—was carefully collected and transferred to a fresh tube. To precipitate RNA, 500 µl isopropanol was added, and samples were placed on ice for 10 min. RNA was pelleted by centrifugation at 12,000 × g for 10 min at 4 °C and washed with 1 ml of 75% ethanol (Sigma-Aldrich). This centrifugation and washing step was repeated, and RNA pellets were air-dried for 5 min at room temperature after removing residual ethanol. RNA was then resuspended in 100 µl DNase/RNase-free water (Thermo Fisher Scientific, Dreieich, Germany) and further purified using the RNeasy Mini Kit.

For this step, 50 µl of RNA obtained from the peqGold TriFast protocol was mixed with 50 µl DNase/RNase-free water. Subsequently, 300 µl RLT buffer containing 1% β-mercaptoethanol (Sigma-Aldrich) and 300 µl of 70% ethanol were added. The mixture was loaded onto a RNeasy column, and washing steps with RW1 and RPE buffers were performed according to the manufacturer’s instructions. Purified RNA was eluted in 25 µl DNase/RNase-free water. Final RNA concentrations were determined using a NanoDrop 2000c spectrophotometer (Thermo Fisher Scientific, Dreieich, Germany).

### Reverse transcription and quantitative PCR

Reverse transcription and quantitative PCR were carried out as previously described [38, 91, 98, 99]. For cDNA synthesis, 500 ng of total RNA from each sample was reverse-transcribed using the iScript cDNA Synthesis Kit (Bio-Rad, Munich, Germany), following the manufacturer’s instructions. Gene expression of *Chat* (Mm01221882_m1), *Slc5a7* (Mm00452075_m1), and *Slc18a3* (Mm00491465_s1) was quantified using TaqMan Gene Expression Assays (Applied Biosystems, Darmstadt, Germany) on a StepOnePlus Real-Time PCR System (Applied Biosystems, Darmstadt, Germany). Each reaction contained 4 ng of cDNA. Threshold cycle (Ct) values for target genes were normalized to *Actb* (Mm00607939_s1) from the same well. Relative expression levels were calculated using the 2^(ΔCt)^ method, where ΔCt = Ct_(*Actb*) – Ct_(target gene). Expression levels of additional genes were assessed using a SYBR Green–based method (AMPLIFYME SYBR Universal Mix; Blirt, Gdansk, Poland). Ct values were normalized to *Actb*, measured in a separate well on the same 96-well plate. Relative expression was again calculated using the 2^(ΔCt)^ method, with ΔCt defined as above. Primer sequences for SYBR-based qPCR are provided in Supplementary Table 3.

### RNA sequencing

Illumina next-generation sequencing libraries, prepared from high-quality input RNA, were analyzed on an Illumina HiSeq 2000 system (Illumina Inc., San Diego, CA, USA) using 50 bp single-end sequencing. Following quality control and adapter trimming using custom software, reads were aligned to the mouse mm10 genome using STAR version 2.4.0 [100]. Gene-level read counts for uniquely aligned reads were determined using featureCounts [101] and subsequently imported into R for differential expression analysis with DESeq2 [102] to identify genes modulated by age, APP overexpression, or an age × APP interaction, with sex included as a covariate. The false discovery rate (FDR) threshold was set at 0.05. For downstream interpretation of differentially expressed genes, Ingenuity Pathway Analysis (IPA) (Ingenuity Systems Inc., Redwood City, CA, USA) was used to perform integrative pathway and functional enrichment analysis. The sample sizes were as follows: APP 12 → 24mo, *n* = 4 mice (2 males and 2 females); APP 6 → 18mo, *n* = 4 mice (2 males and 2 females); 24mo control, *n* = 3 mice (2 males and 1 female); 18mo control, *n* = 4 mice (2 males and 2 females).

### Statistics

Statistical analyses were performed using GraphPad Prism (version 10.0.2; GraphPad Software, La Jolla, CA, USA) and IBM SPSS Statistics (version 23; IBM, Armonk, NY, USA). Unless stated otherwise, data were analyzed using two-way analysis of variance (ANOVA) with the between-subjects factors age (24-month-old vs. 18-month-old) and APP overexpression (APP overexpression vs. APP expression suppressed by doxycycline). Fisher’s LSD test was used for posthoc analyses, where appropriate. Statistical significance was defined as follows: * *p* < 0.05, ** *p* < 0.01, *** *p* < 0.001, and **** *p* < 0.0001.

## Supplementary Information

Supplementary Material 1.

Supplementary Material 2.

Supplementary Material 3.

Supplementary Material 4.

## Data Availability

Raw RNA sequencing files and normalized count data from Fig. 6 and Supplementary Fig. 7 are available in the Gene Expression Omnibus (GEO) under accession number GSE271806.
