# Supplementary material for: Selective vulnerability of the aging cholinergic system to amyloid pathology revealed by induced APP overexpression
Source: J Neuroinflammation. 2026 Jan 7;23:39. doi: 10.1186/s12974-025-03682-2 (PMC12849525; doi:10.1186/s12974-025-03682-2)
Supplement: Supplementary file 1 — Supplementary Material 1. [file 12974_2025_3682_MOESM1_ESM.pdf]

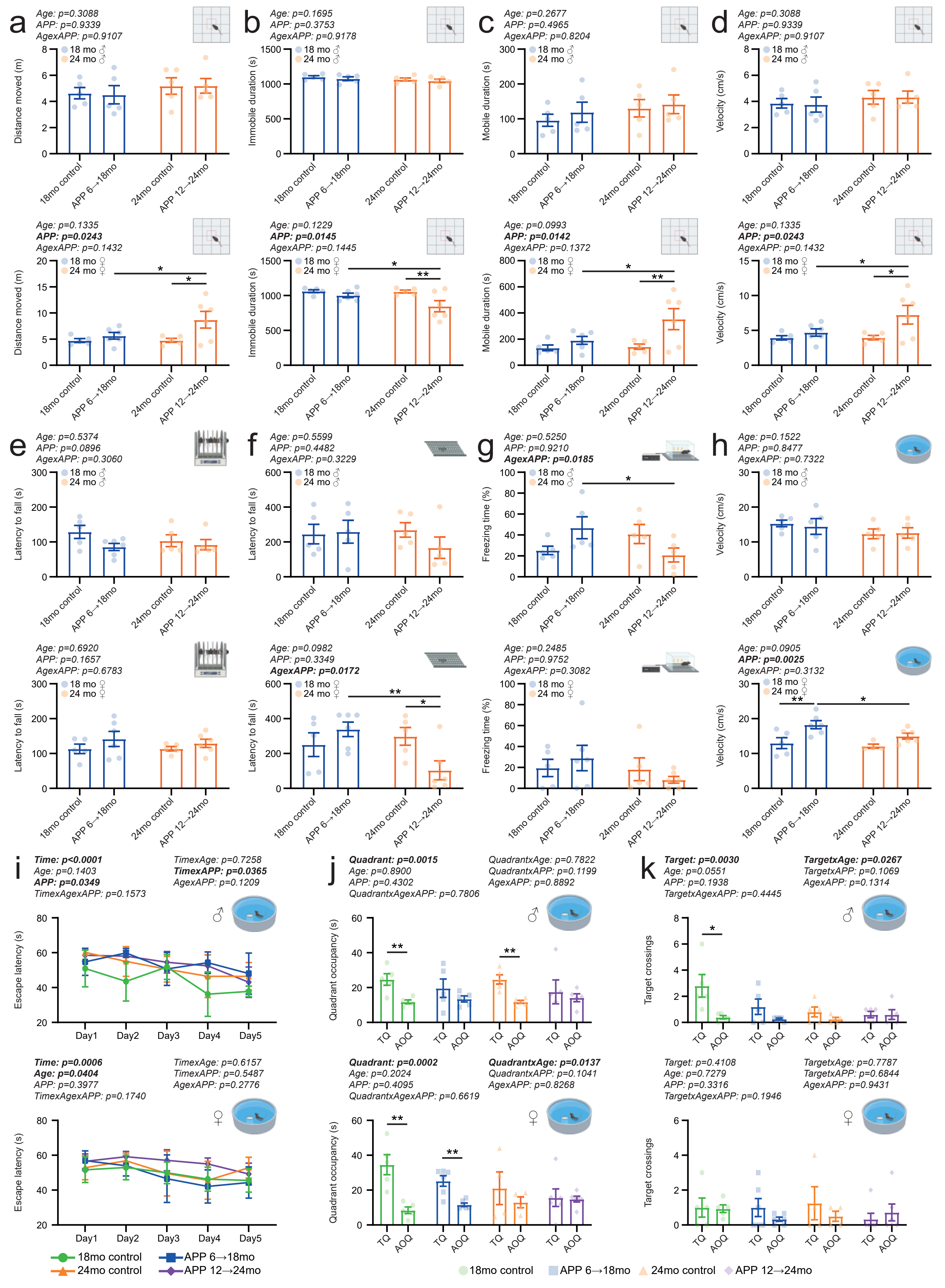

Supplementary Figure 1

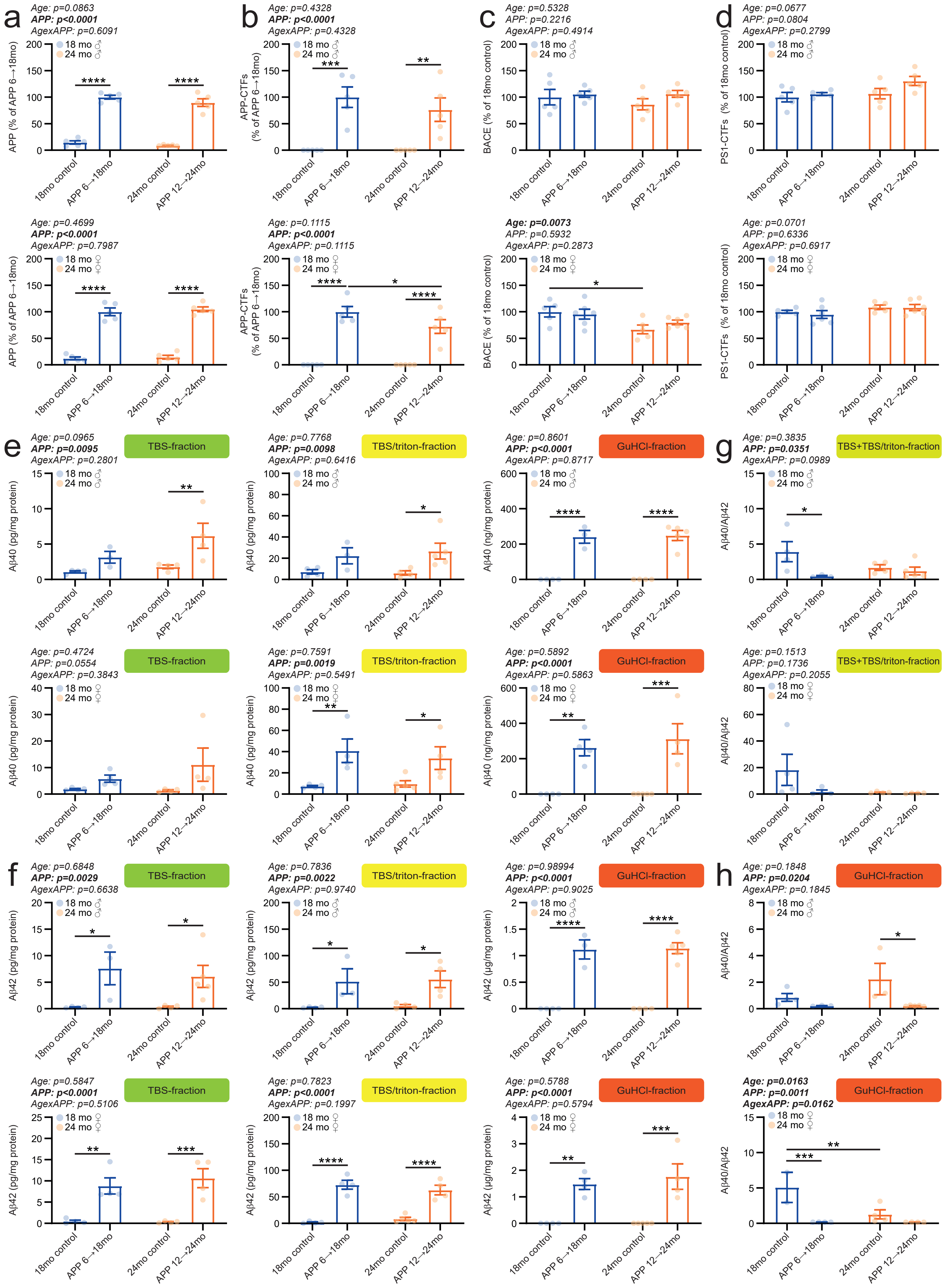

Supplementary Figure 2

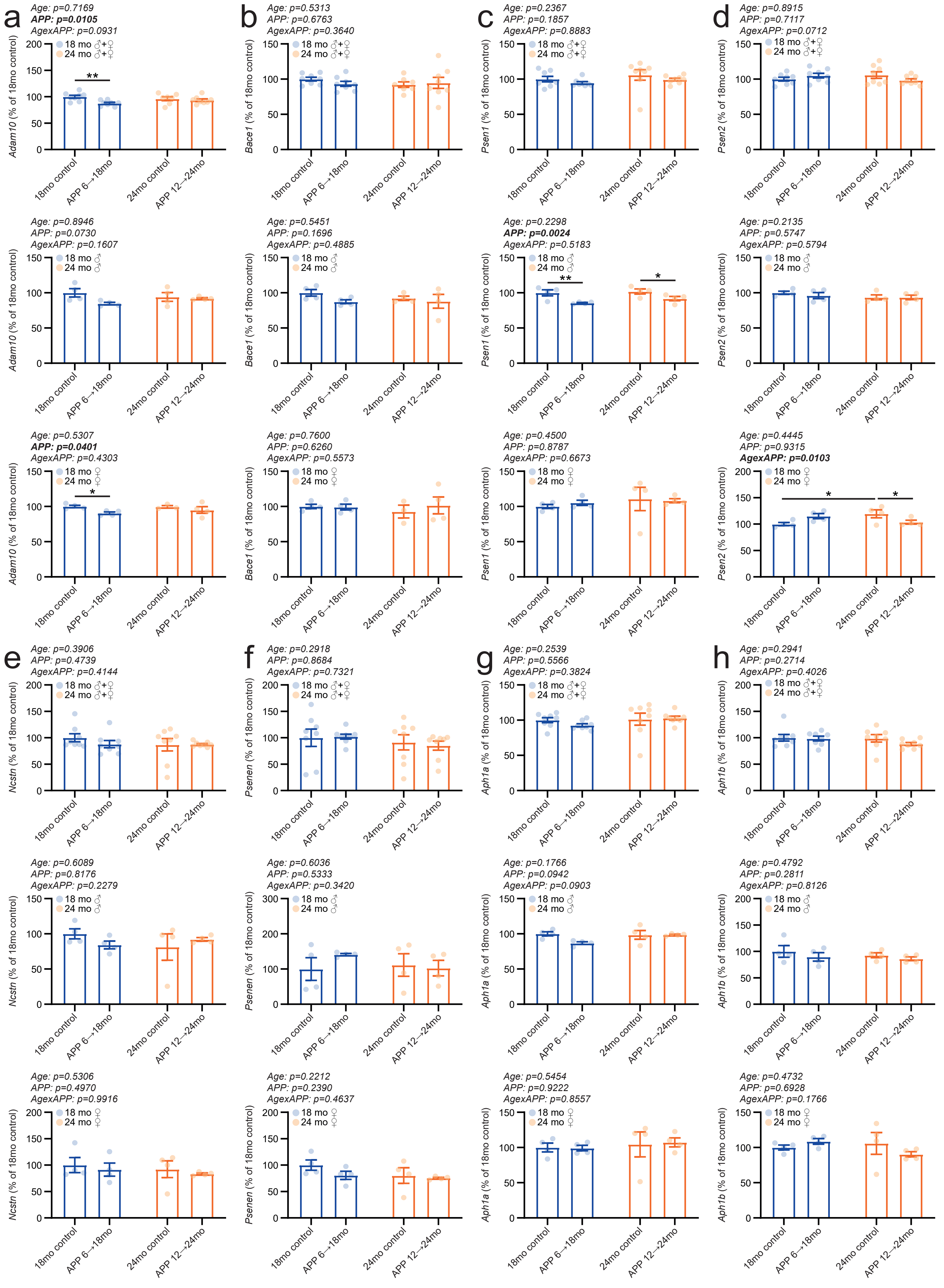

Supplementary Figure 3

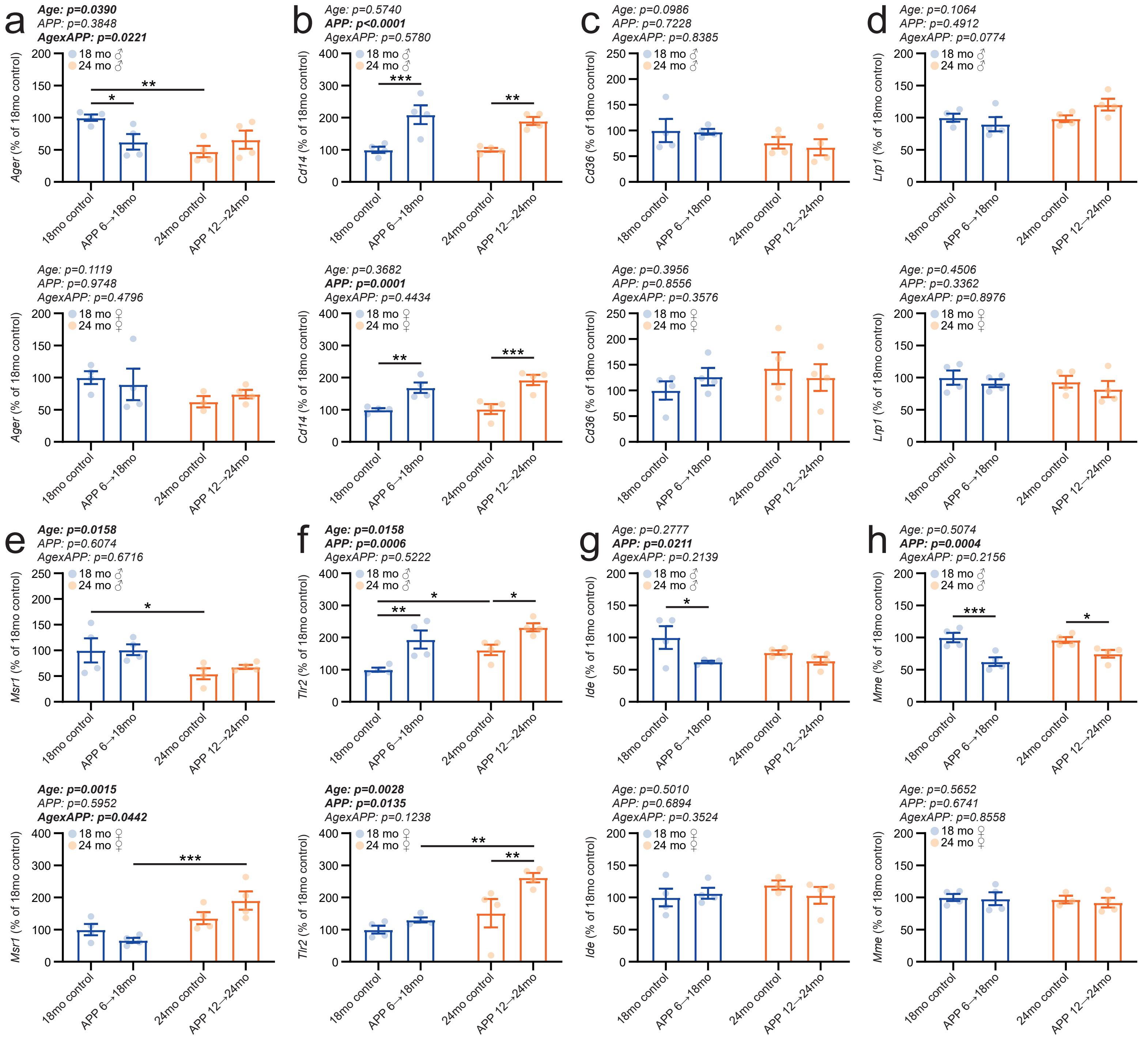

Supplementary Figure 4

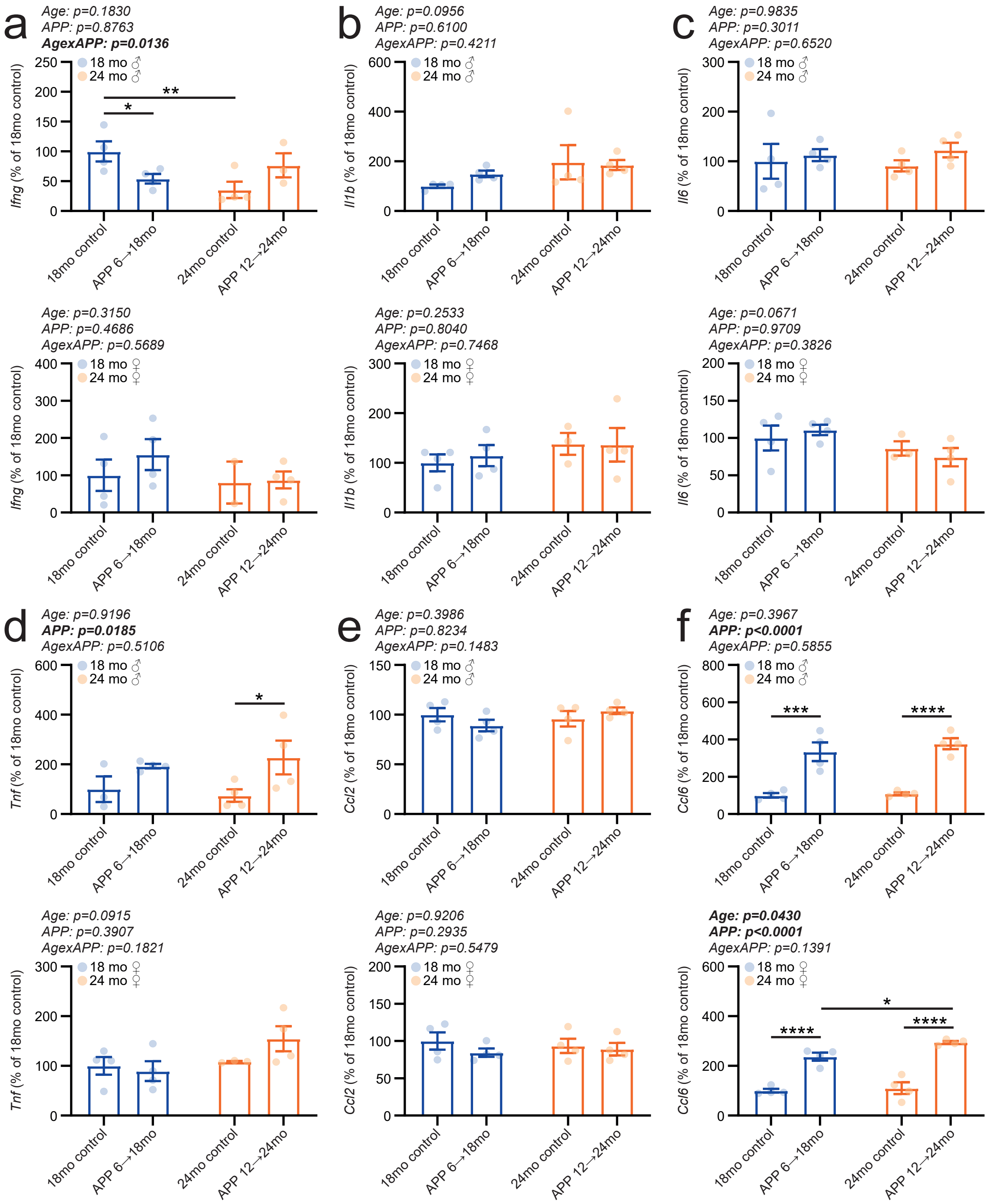

Supplementary Figure 5

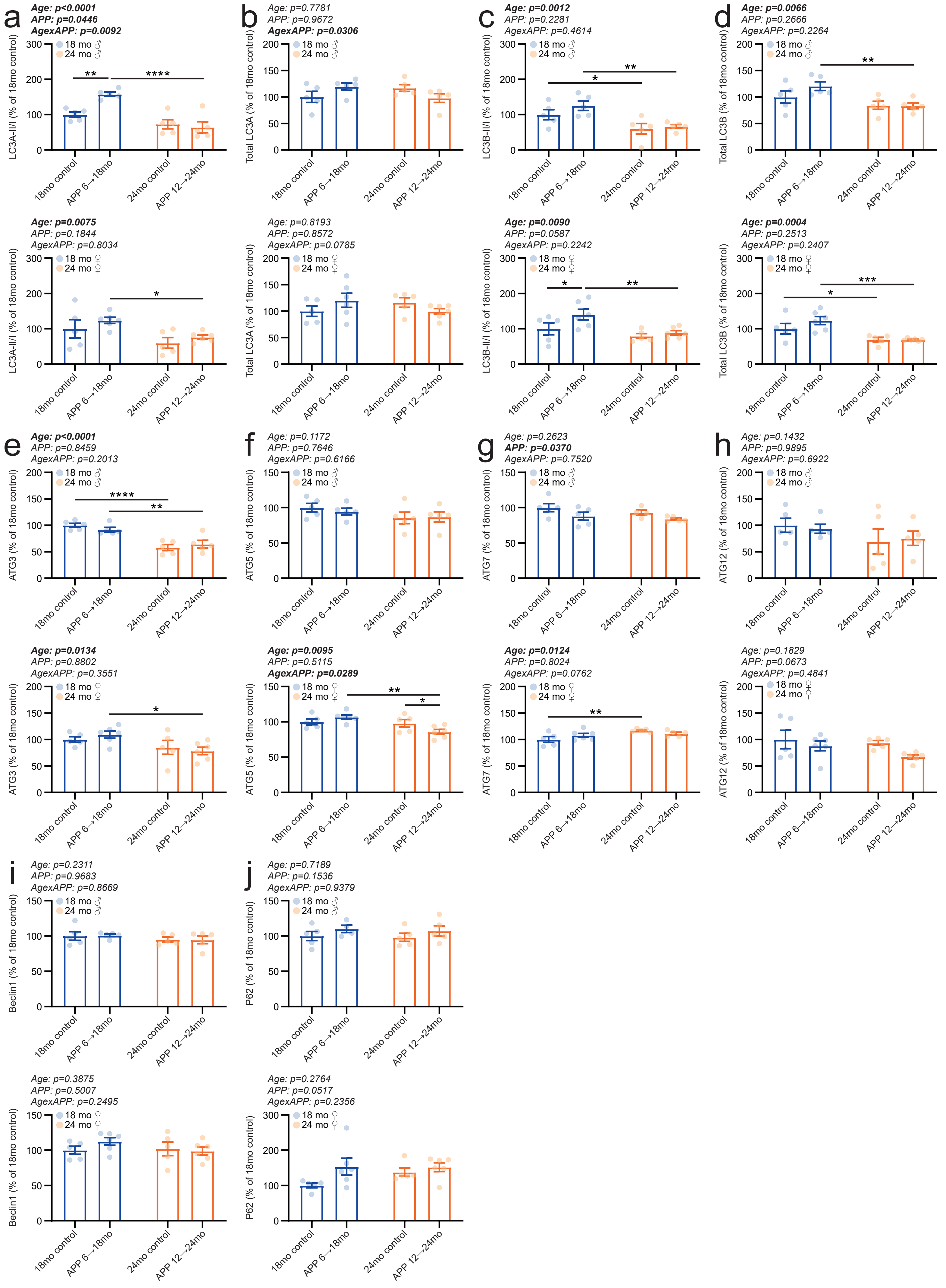

Supplementary Figure 6

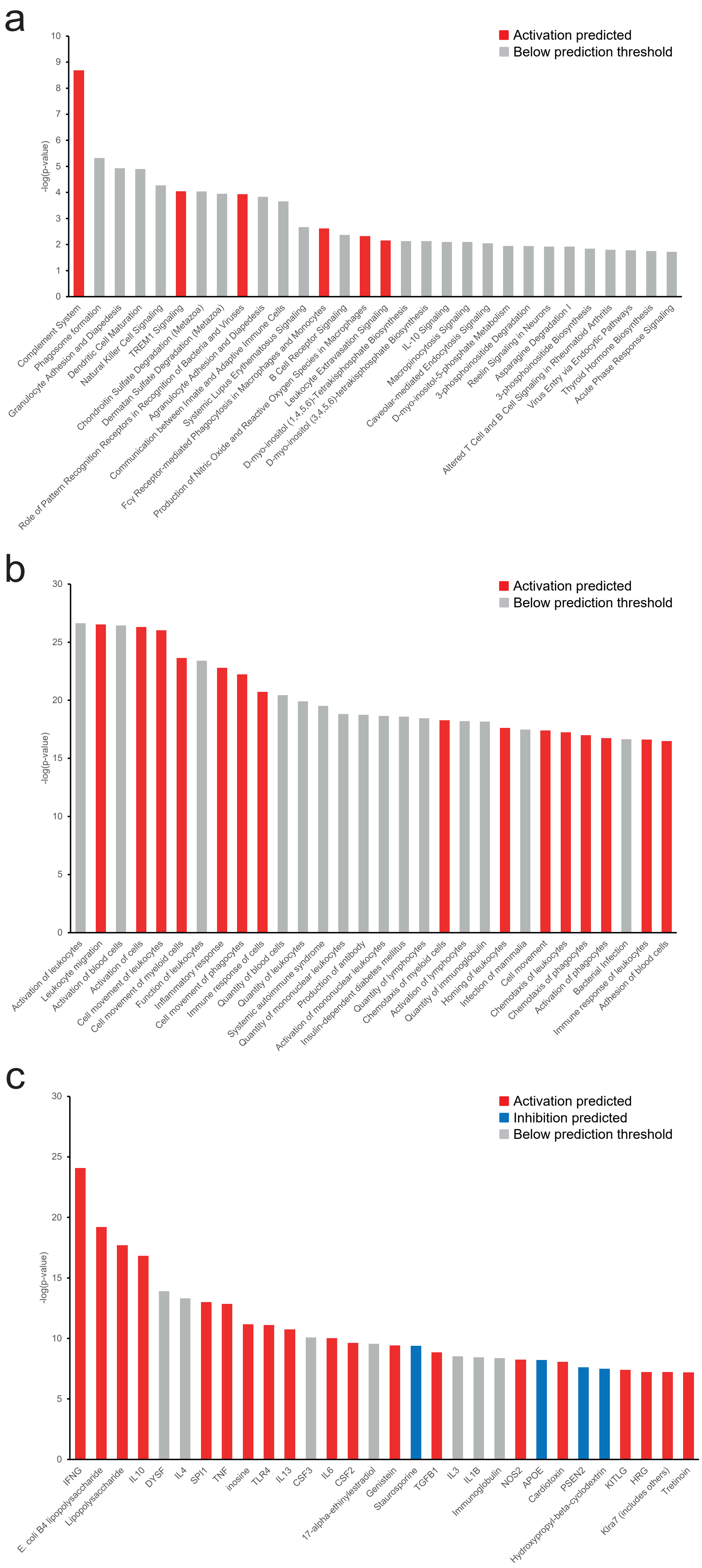

Supplementary Figure 7

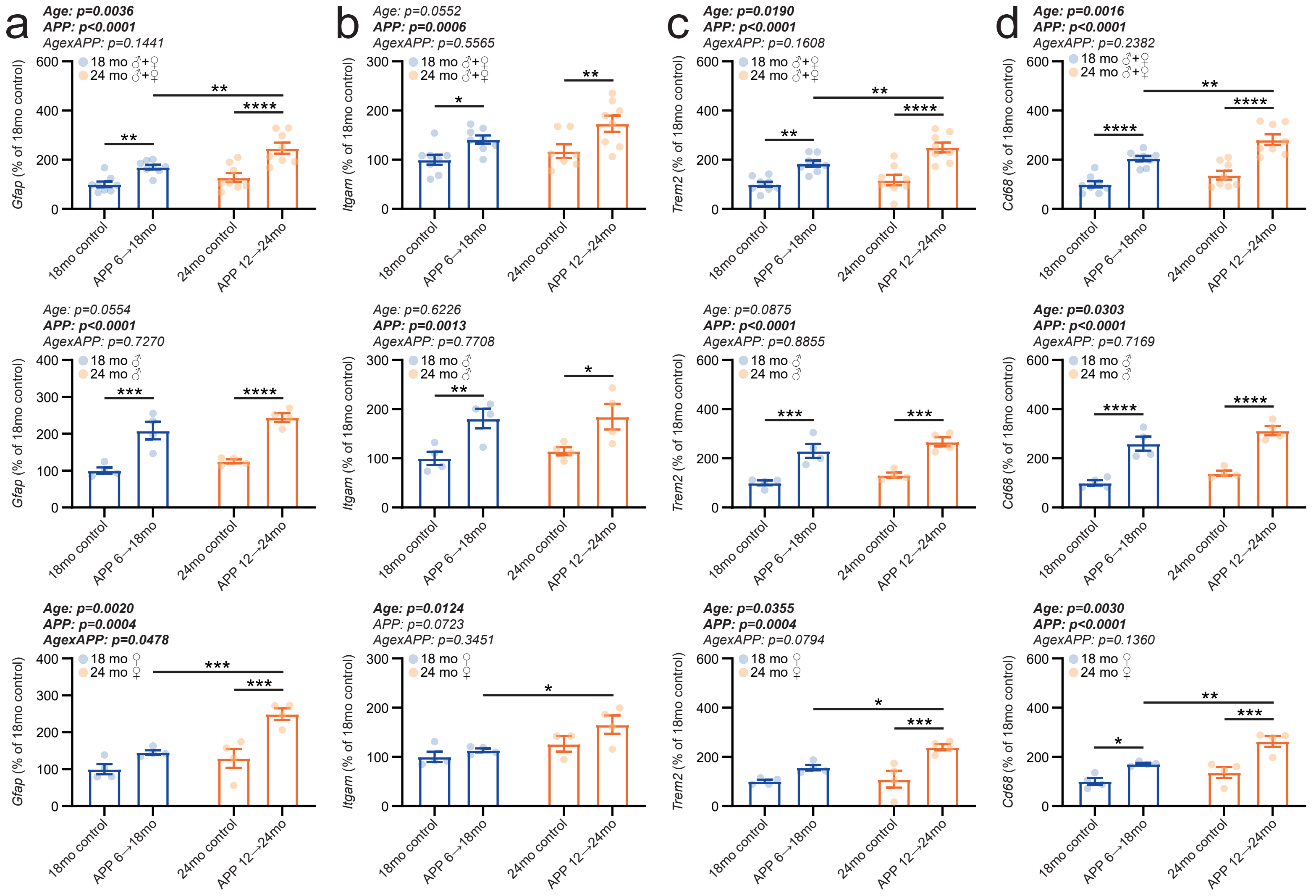

Supplementary Figure 8

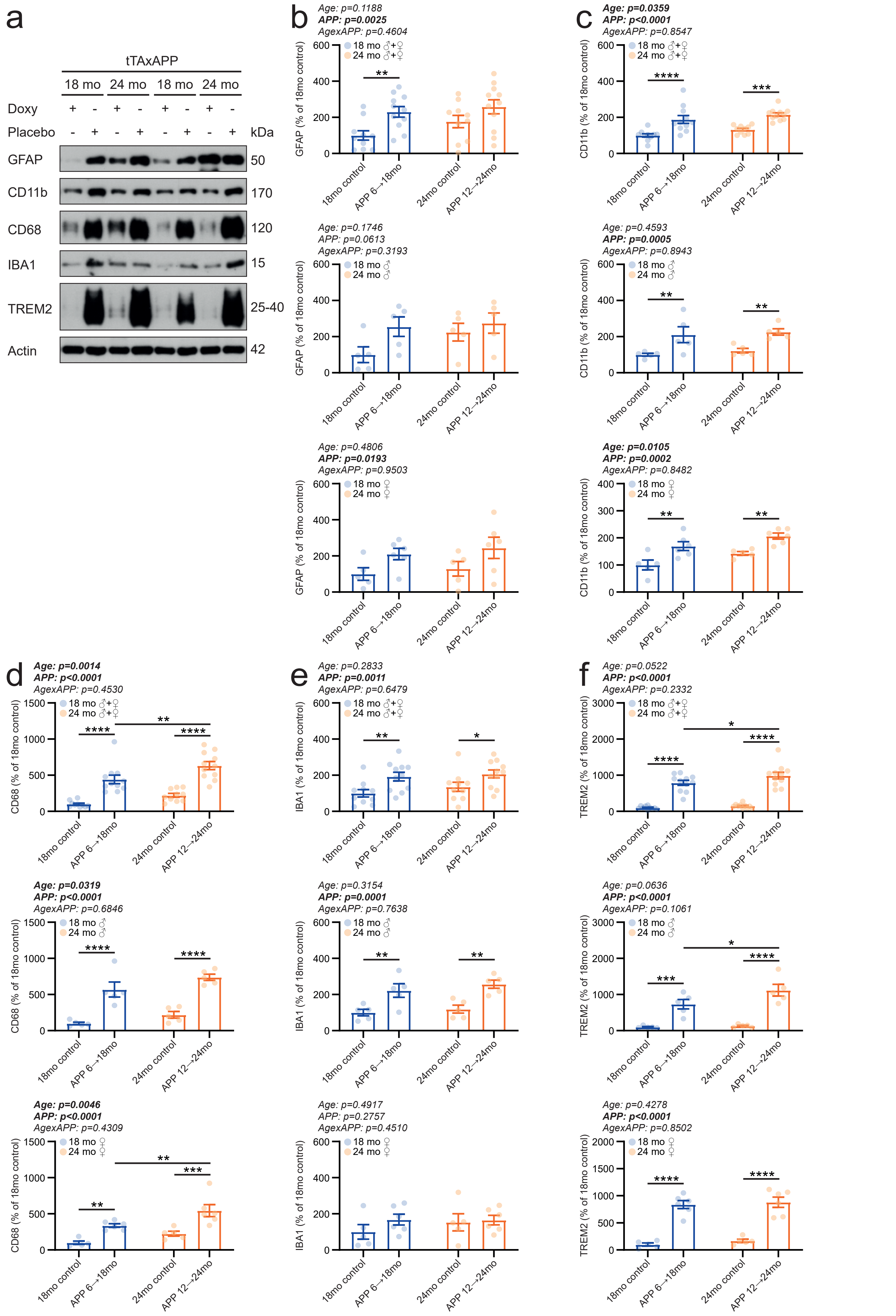

Supplementary Figure 9

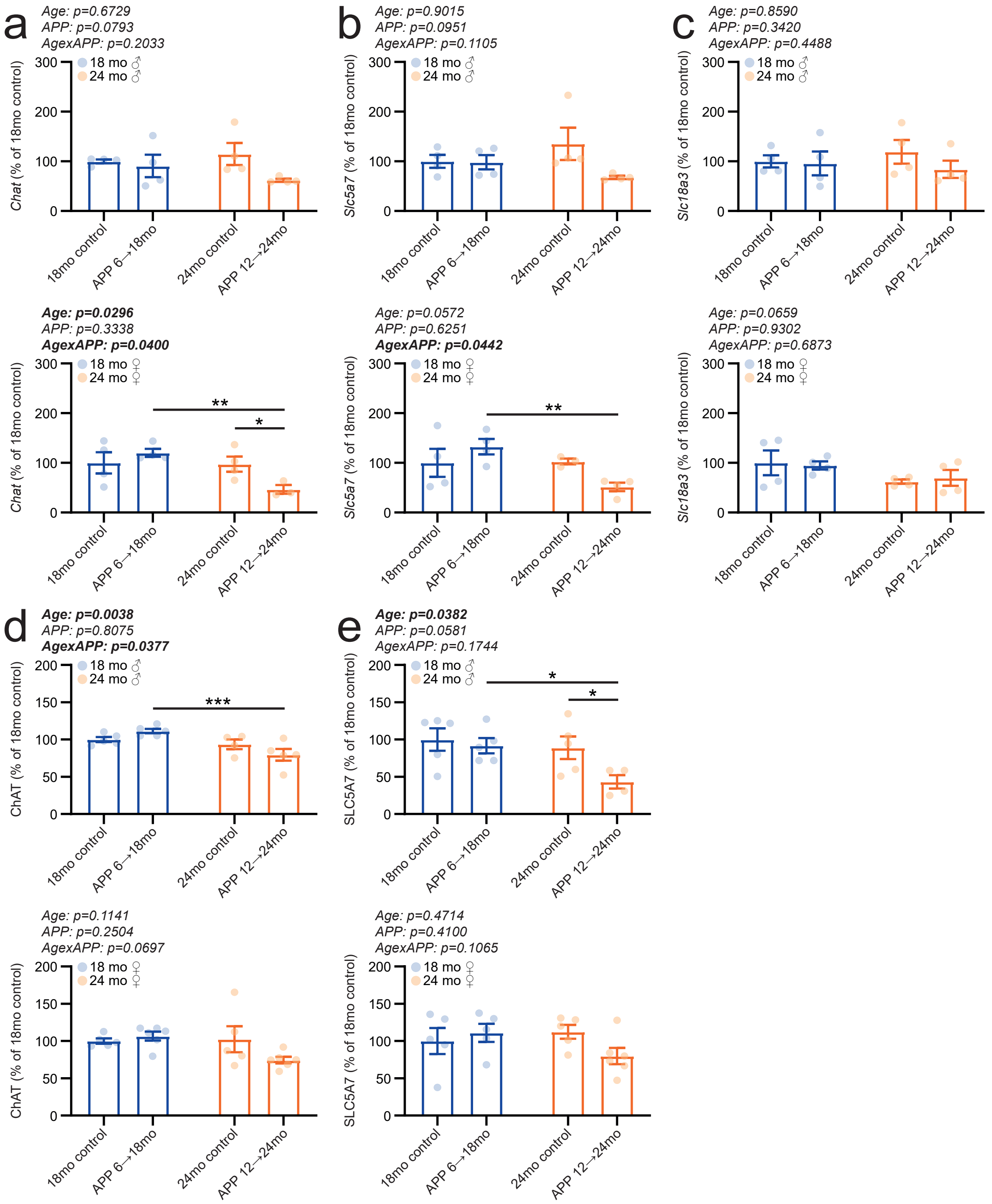

Supplementary Figure 10

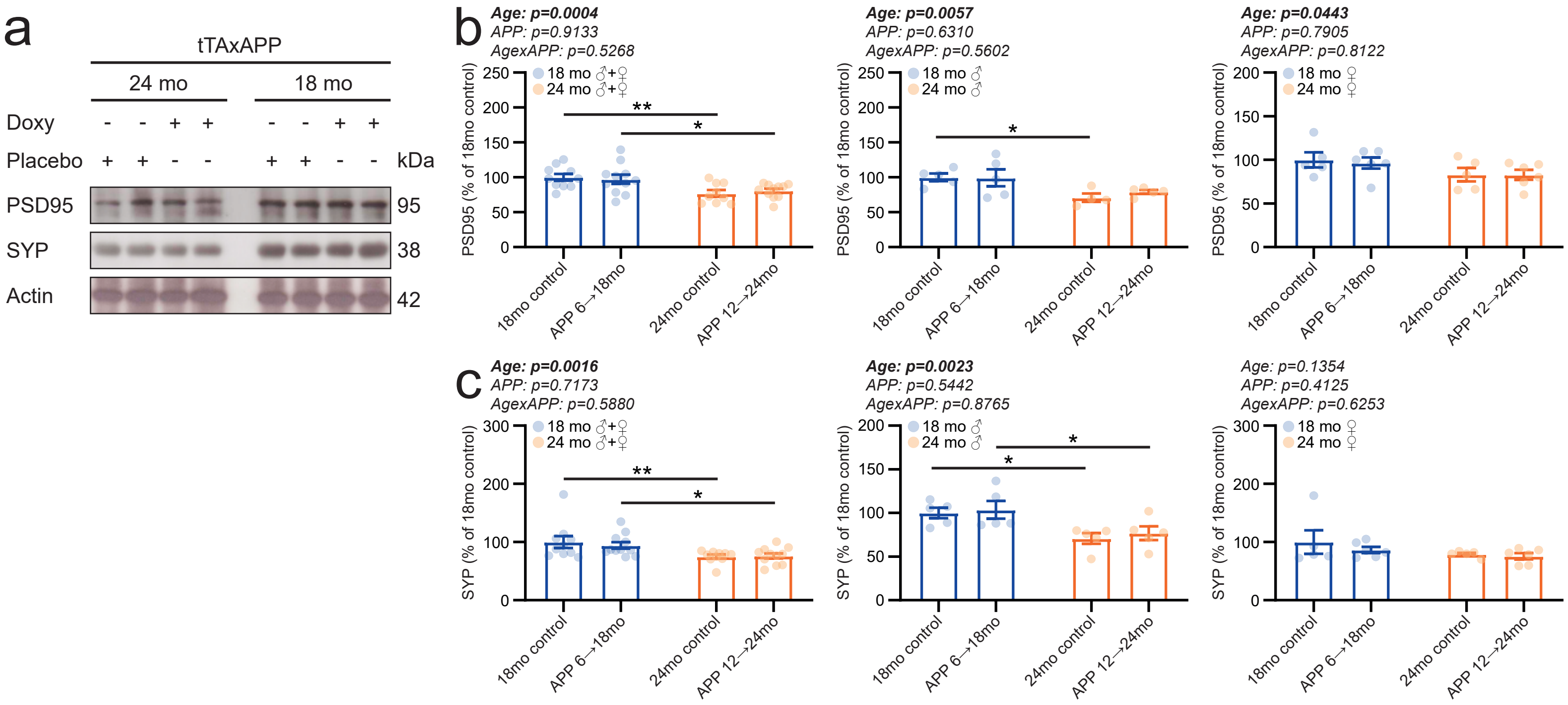

Supplementary Figure 11
