# Supplementary material for: Selective vulnerability of the aging cholinergic system to amyloid pathology revealed by induced APP overexpression
Source: J Neuroinflammation. 2026 Jan 7;23:39. doi: 10.1186/s12974-025-03682-2 (PMC12849525; doi:10.1186/s12974-025-03682-2)
Supplement: Supplementary file 2 — Supplementary Material 2. [file 12974_2025_3682_MOESM2_ESM.pdf]

Selective Vulnerability of the Aging Cholinergic System to Amyloid Pathology Revealed by Induced APP Overexpression

Kan Xie[1], Devon Ryan[1$], Susanne Schröder[1], Lena Freund[1&], Stefan Bonn[2], Yu Zhou[3] and Dan Ehninger[1]*

*Correspondence should be addressed to: Dan.Ehninger@dzne.de

[1]Translational Biogerontology Lab, German Center for Neurodegenerative Diseases (DZNE), Venusberg-Campus 1/99, 53127 Bonn, Germany

[2]Institute of Medical Systems Bioinformatics, Center for Biomedical AI (bAIome), Center for Molecular Neurobiology (ZMNH), Center for Translational Immunology (HCTI), German Center for Child and Youth Health (DZKJ), University Medical Center Hamburg-Eppendorf, 20246 Hamburg, Germany

[3]School of Life Sciences and Health, University of Health and Rehabilitation Sciences, Qingdao, Shandong 266021, China

[$]Present address: Genedata AG, Margarethenstr. 38, 4053 Basel, Switzerland

[&]Present address: Fraunhofer Institute for Molecular Biology and Applied Ecology IME, Forckenbeckstr. 6, 52074 Aachen, Germany

Supplementary Table 1. Comparison of APP-associated differentially expressed genes (DEGs) uncovered in the present study with a systematic meta-analysis of human AD transcriptomic datasets

Gene symbol	DEG in the present study	DEG in human AD by Yuen et al., 2020
4632428N05Rik	True	False
Adam33	True	True
Anxa4	True	False
App	True	False
Arl11	True	True
Asb10	True	True
Aspg	True	True
Atp13a4	True	False
B430306N03Rik	True	False
C1qa	True	True
C1qb	True	False
C1qc	True	True
C3ar1	True	True
C4b	True	True
Cckbr	True	True
Ccl3	True	False
Ccl4	True	True
Ccl6	True	False
Cd14	True	True
Cd180	True	True
Cd37	True	True
Cd44	True	True
Cd52	True	False
Cd53	True	True
Cd68	True	False
Cd72	True	False
Cd84	True	True
Cd9	True	False
Clec7a	True	True
Creg1	True	False
Csf1r	True	True
Csf3r	True	True
Cst7	True	True
Ctsd	True	False
Ctsl	True	True
Ctss	True	True
Ctsz	True	False
Cttnbp2	True	False
Cx3cr1	True	False
Cxcl5	True	False
Dclre1b	True	True
Derl1	True	True
Dusp14	True	True
Fam46c	True	False
Fcer1g	True	True

Fcgr2b	True	True
Fcgr3	True	False
Fcrls	True	False
Fgr	True	True
Flnc	True	True
Gfap	True	True
Ggta1	True	True
Glipr2	True	True
Gpr183	True	True
Gpr65	True	True
Grn	True	False
Gusb	True	False
Hexa	True	False
Hexb	True	True
Irf5	True	True
Irf8	True	True
Itgax	True	True
Itgb2	True	True
Kirrel2	True	True
Klhl6	True	True
Krt20	True	False
Lag3	True	False
Lair1	True	True
Laptm5	True	True
Lcp1	True	True
Lgals3bp	True	False
Lilrb4	True	True
LOC101056001	True	False
Ly86	True	True
Mamdc2	True	False
Man2b1	True	False
Milr1	True	False
Mpeg1	True	False
Msn	True	True
Myo1f	True	True
NA	True	False
Ncf1	True	False
Niacr1	True	False
Nrsn1	True	True
Olfml3	True	True
Oprk1	True	True
Osmr	True	True
P2ry13	True	False
Pdcd1	True	True
Plek	True	True
Plxdc2	True	True
Pou3f3os	True	False
Prnp	True	False
Ptpn6	True	True
Ptprc	True	True
Ptprr	True	True
Pycard	True	True
R3hdm1	True	True

Rac2	True	True
Rdh11	True	True
Renbp	True	True
S100a16	True	False
Samsn1	True	True
Serpina3n	True	False
Slamf9	True	False
Slc11a1	True	True
Slc14a1	True	True
Slc15a2	True	True
Slc15a3	True	True
Slc5a7	True	True
St14	True	True
St6galnac2	True	False
Stac3	True	False
Stard4	True	False
Syngr2	True	False
Tbxas1	True	True
Tlr13	True	False
Tlr2	True	True
Tmem176a	True	True
Tnfaip8l2	True	False
Tnfrsf1a	True	True
Tnfsf8	True	False
Trem2	True	True
Treml2	True	False
Trpm1	True	True
Tyrobp	True	True
Wfdc17	True	False

Supplementary Table 2. Gene expression levels of mouse brain cell lineage markers

Differential expression analysis was conducted to compare brain transcriptomic profiles between APP 6→18mo (2 males and 2 females), APP 12→24mo (2 males and 2 females), and age-matched controls (2 male and 1 female 18-month-old controls; 2 male and 2 female 24-month-old controls). A false discovery rate (FDR) threshold of 0.05 was applied. Statistically significant comparisons are highlighted in bold. NA = p-value not available.

Cell type	Specific cell type	Gene	P-value (Age)	P-value (APP)	P-value (AgexAPP)
Glia	Astrocytes	*Aldh1l1*	0.9998	0.9492	0.9999
		Aldoa	0.9998	0.8978	0.9999
		Aldob	0.9998	0.9525	0.9999
		Aldoc	0.9998	0.8041	0.9999
		Aqp4	0.9998	0.1118	0.9999
		Coro1a	0.9998	0.8978	0.9999
		Gfap	0.9998	**<0.0001**	0.9999
		Igfbp3	0.9998	0.9924	0.9999
		S100b	0.9998	0.5919	0.9999
	Microglia	*Aif1*	0.9998	0.6631	0.9999
		C1qA	0.9998	**<0.0001**	0.9999
		C1qB	0.9998	**0.0002**	0.9999
		Cd14	0.9998	**<0.0001**	0.9999
		Cd40	NA	0.7956	0.9999
		Cd68	0.9998	**<0.0001**	0.9999
		Cst3	0.9998	0.8654	0.9999
		Cx3cr1	0.9998	**0.0123**	0.9999
		Emr1	0.9998	0.2944	0.9999
		Itgam	0.9998	0.0901	0.9999
		Nos2	0.8248	0.9369	0.9999
		P2ry12	0.9998	0.3255	0.9999
		Ptprc	0.9998	**0.0092**	0.9999
		Pycard	0.9998	**0.0433**	0.9999
		Spi1	0.9998	0.0647	0.9999
		Tlr2	0.9998	**0.0139**	0.9999
		Tmem119	0.9998	0.9984	0.9999
	Oligodendrocytes	*Cldn11*	0.9998	0.5186	0.9999
		Cnp	0.9998	0.7725	0.9999
		Cspg4	0.9998	0.9866	0.9999
		Galc	0.9998	0.8412	0.9999
		Mag	0.9998	0.8212	0.9999
		Mbp	0.9998	0.8436	0.9999
		Mog	0.9998	0.8428	0.9999

		Olig1	0.9998	0.9966	0.9999
		Olig2	0.9998	0.9866	0.9999
		Pdgfra	0.9998	0.8731	0.9999
	**Radial glia**	*Fabp7*	0.9998	0.9905	0.9999
		Tnc	0.9998	0.9866	0.9999
		Vim	0.9998	0.2078	0.9999
**Neurons**	**Neural progenitor cells**	*Ascl1*	0.9998	0.9009	0.9999
		Cntnap1	0.9998	0.9966	0.9999
		Cdh1	0.9998	0.7956	0.9999
		Cdh2	0.9998	0.8654	0.9999
		Fut4	NA	0.9785	0.9999
		Msi1	0.9998	0.9782	0.9999
		Msi2	0.9998	0.9866	0.9999
		Nes	0.9998	0.9492	0.9999
		Notch1	0.9998	0.9534	0.9999
		Otx2	0.9998	0.9292	0.9999
		Pax3	NA	0.5508	0.9999
		Pax6	0.9998	0.9083	0.9999
		Smarca4	0.9998	0.8640	0.9999
		Sox1	0.9998	0.9994	0.9999
		Sox2	0.9998	0.8519	0.9999
	**Immature neurons**	*Dcx*	0.9998	0.9429	0.9999
		Eno2	0.9998	0.7846	0.9999
		Ncam1	0.9998	0.8654	0.9999
		Neurod1	0.9998	0.7824	0.9999
		Stmn1	0.9998	0.9045	0.9999
		Tbr1	0.9998	0.9740	0.9999
	**Mature neurons**	*Dlg4*	0.9998	0.5281	0.9999
		Ina	0.9998	0.9080	0.9999
		Map2	0.9998	0.9898	0.9999
		Mapt	0.9998	0.9510	0.9999
		Nefh	0.9998	0.7970	0.9999
		Nefl	0.9998	0.9357	0.9999
		Nefm	0.9998	0.9084	0.9999
		Nrp1	0.9998	0.8505	0.9999
		Rbfox3	0.9998	0.8978	0.9999
		Syp	0.9998	0.5319	0.9999
		Thy1	0.9998	0.8695	0.9999
		Tubb3	0.9998	0.9984	0.9999
		Uchl1	0.9998	0.9866	0.9999
	**Cholinergic neurons**	*Ache*	0.9998	0.9966	0.9999
		Chat	**0.0003**	0.2561	**0.0241**
		Slc5a7	**0.0004**	**0.0068**	**0.0034**
	**Dopaminergic neurons**	*Dbh*	0.9998	0.9168	0.9999
		Foxa2	NA	NA	0.9999
		Kcnj3	0.9998	0.1830	0.9999

		Lmx1b	NA	0.9587	0.9999
		Nr4a2	0.9998	0.8519	0.9999
		Ppp1r1b	0.9998	0.9686	0.9999
		Slc6a2	0.9998	0.9779	0.9999
		Slc6a3	0.9998	0.8702	0.9999
		Th	0.9998	0.4542	0.9999
	**GABAergic neurons**	*Adora2a*	0.9998	0.9714	0.9999
		Calb1	0.9998	0.8686	0.9999
		Calb2	0.9998	0.9586	0.9999
		Gabbr1	0.9998	0.8519	0.9999
		Gabbr2	0.9514	0.9774	0.9999
		Gad1	0.9998	0.9133	0.9999
		Gad2	0.9998	0.8612	0.9999
		Pvalb	0.9998	0.9309	0.9999
		Penk	0.4800	0.8395	0.9999
		Slc6a1	0.9998	0.8702	0.9999
		Slc32a1	0.9998	0.9347	0.9999
		Sst	0.9998	0.8519	0.9999
	**Glutamatergic neurons**	*Gls*	0.9998	0.9579	0.9999
		Glul	0.9998	0.9857	0.9999
		Grin1	0.9998	0.9587	0.9999
		Grin2a	0.9998	0.9871	0.9999
		Grin2b	0.9998	0.9083	0.9999
		Slc17a6	0.9998	0.9181	0.9999
		Slc17a7	0.9998	0.5909	0.9999
	**Serotonergic neurons**	*Fev*	NA	NA	0.9999
		Slc6a4	NA	0.9391	0.9999
		Tph1	NA	NA	0.9999
		Tph2	0.9998	0.9308	0.9999

Supplementary Table 3. Primers used for qPCR-based analyses

Gene	Primer forward	Primer reverse
Actb	CCCTGAAGTACCCCATTGAAC	CCATGTCGTCCCAGTTGGTAA
Adam10	TGATGGTGTTCTTGGTCTGG	CTTGCTTTTCTCACATATTCCCC
Ager	ACCCATCCTACCTTCTCCTG	GAGCGACTATTCCACCTTCAG
Aph1a	CCAGTATGGCCTCCTGATTT	TGATGCTAAGCCCTCATCTG
Aph1b	TTGGTGTCTCTCCTGCTGTC	AGCCTGAACAGCTCTTGGAT
Bace1	TTGTAGGGCTAGGGATGGTC	CCTAACCCTGCTGGATGAAT
Ccl2	AAGAGATCAGGGAGTTTGCT	CTGCCTCCATCAACCACTTT
Ccl6	TCTTTATCCTTGTGGCTGTCC	ATGGGATCTGTGTGGCATAAG
Cd14	TTTAACTCTGGCGTAGTCACC	GACCCTCAGAAACCAGGAG
Cd36	GCGACATGATTAATGGCACAG	GATCCGAACACAGCGTAGATAG
Cd68	GTTACTCTCCTGCCATCCTTC	GCAGGGTTATGAGTGACAGTTG
Gfap	GAAAACCGCATCACCATTCC	CTTAATGACCTCACCATCCCG
Ide	CCCGACTTGATAGACATGGTTC	CGGTCAGTTTTCCCTTCAAATG
Ifng	CTTTGGACCCTCTGACTTGAG	TCAATGACTGTGCCGTGG
Il1b	GAAGAAGAGCCCATCCTCTG	TCATCTCGGAGCCTGTAGTG
Il6	AGTCCGGAGAGGAGACTTCA	ATTTCCACGATTTCCCAGAG
Itgam	CATCCCATGACCTTCCAAGAG	GTGCTGTAGTCACACTGGTAG
Lrp1	GGGAAGTGATGGGAAGTCTTG	GGATTCATAAGGTTCTCGATGGG
Mme	GATGAGTGGATAAGTGGAGCAG	CAATGAGTTGGATTGCTGAGC
Msr1	GGGAACACTCACAGACACTG	CCCGATCACCTTTAACACCTG
Ncstn	GTACTGGCAGGATTGTGTGG	ATCTGATGAGTGGCGTTGAG
Psen1	CTGGTTGAAACAGCTCAGGA	TTCTTGGGTACCCTCCTTTG
Psen2	ACGCTGTGTATGATCGTGGT	TGTTAAGCACGGAGTTGAGG
Psenen	GGTTCTTCAGAGAGGCGTTC	GATCACCCAGAAGAGGAAGC
Tlr2	ACAACTTACCGAAACCTCAGAC	ACCCCAGAAGCATCACATG
Tnf	CTTCTGTCTACTGAACTTCGGG	CAGGCTTGTCACTCGAATTTTG
Trem2	GCTTGGTCATCTCTTTTCTGC	GTTGAGGGCTTGGGACAG

Supplementary Figure 1. Analyses of behavioral and cognitive assessments stratified by sex.

(**a**) Distance traveled, (**b**) immobile duration, (**c**) mobile duration, and (**d**) movement speed recorded in the open field test (5 male and 5 female 18mo controls; 5 male and 6 female APP 6→18mo; 5 male and 5 female 24mo controls; 5 male and 6 female APP 12→24mo). (**e**) Latency to fall on the accelerating rotarod (5 male and 5 female 18mo controls; 6 male and 6 female APP 6→18mo; 5 male and 5 female 24mo controls; 5 male and 6 female APP 12→24mo). (**f**) Latency to fall on the inverted screen test (5 male and 5 female 18mo controls; 5 male and 6 female APP 6→18mo; 5 male and 5 female 24mo controls; 5 male and 6 female APP 12→24mo). (**g**) Percent time spent freezing during the test session in a contextual fear conditioning paradigm (5 male and 5 female 18mo controls; 5 male and 6 female APP 6→18mo; 5 male and 5 female 24mo controls; 5 male and 6 female APP 12→24mo). (**h**) Swim speed and (**i**) escape latency during the training phase of the Morris Water Maze (MWM) (5 male and 5 female 18mo controls; 5 male and 6 female APP 6→18mo; 5 male and 4 female 24mo controls; 5 male and 6 female APP 12→24mo). (**j**) Time spent in the target quadrant (TQ) vs. the average of all other quadrants (AOQ), and (**k**) number of platform crossings during the MWM probe trial. The panels (**i-k**) were analyzed by three-way ANOVA. Individual data points and group means ± S.E.M. are shown. * $p<0.05$, ** $p<0.01$.

Supplementary Figure 2. Levels of APP and brain amyloid burden stratified by sex.

Quantification of (**a**) full-length APP and (**b**) APP C-terminal fragments (APP-CTFs) in 18mo control (5 males and 5 females), APP 6→18mo (5 males and 5 females), 24mo control (5 males and 5 females), and APP 12→24mo (5 males and 5 females). Quantification of (**c**) BACE

and (**d**) PS1 C-terminal fragments (PS1-CTFs) in 18mo control (5 males and 5 females), APP 6→18mo (5 males and 6 females), 24mo control (5 males and 5 females), and APP 12→24mo (5 males and 6 females). ELISA-based measurements of (**e**) Aβ40 and (**f**) Aβ42 levels in TBS-fraction (4 male and 4 female 18mo controls; 3 male and 4 female APP 6→18mo; 4 male and 4 female 24mo controls; 4-5 male and 4 female APP 12→24mo), TBS-triton-fraction (4 male and 4 female 18mo controls; 3 male and 4 female APP 6→18mo; 4 male and 5 female 24mo controls; 4-5 male and 4 female APP 12→24mo), and GuHCl-fraction (4 male and 4 female 18mo controls; 3 male and 4 female APP 6→18mo; 4 male and 5 female 24mo controls; 5 male and 4 female APP 12→24mo). (**g**) Aβ40/Aβ42 ratio calculated for TBS and TBS + Triton X-100 soluble Aβ species. (**h**) Aβ40/Aβ42 ratio for GuHCl-soluble higher-order Aβ aggregates. Individual data points and group means ± S.E.M. are shown. * p<0.05, ** p<0.01, *** p<0.001, **** p<0.0001.

Supplementary Figure 3. Gene expression levels of α-, β-, and γ-secretase components measured by qPCR.

mRNA levels of (**a**) *Adam10*, (**b**) *Bace1*, (**c**) *Psen1*, (**d**) *Psen2*, (**e**) *Ncstn*, (**f**) *Psenen*, (**g**) *Aph1a*, and (**h**) *Aph1b* were measured in brain tissue from APP 6→18mo (4 males and 4 females), APP 12→24mo (4 males and 4 females), and age-matched control mice (4 male and 4 female 18mo controls; 4 male and 3-4 female 24mo controls). Individual data points as well as mean ± S.E.M. are shown. * p<0.05, ** p<0.01.

Supplementary Figure 4. Sex-stratified analyses of gene transcription of receptors and enzymes critical for Aβ clearance and degradation.

mRNA levels of (**a**) *Ager*, (**b**) *Cd14*, (**c**) *Cd36*, (**d**) *Lrp1*, (**e**) *Msr1*, (**f**) *Tlr2*, (**g**) *Ide*, and (**h**) *Mme* were measured in the brains of APP 6→18mo (4 males and 4 females), APP 12→24mo (4

males and 4 females), and age-matched control animals (4 male and 4 female 18mo controls; 4 male and 3-4 female 24mo controls). Individual data points and group means ± S.E.M. are shown. * p<0.05, ** p<0.01, *** p<0.001.

Supplementary Figure 5. mRNA levels of inflammatory cytokines and chemokines stratified by sex.

mRNA levels of (**a**) *Ifng*, (**b**) *Il1b*, (**c**) *Il6*, (**d**) *Tnf*, (**e**) *Ccl2*, and (**f**) *Ccl6* were measured in the brains of APP 6→18mo (4 males and 4 females), APP 12→24mo (3-4 males and 4 females), and age-matched control animals (3-4 male and 4 female 18mo controls; 4 male and 2-4 female 24mo controls). Individual data points and group means ± S.E.M. are shown. * p<0.05, ** p<0.01, *** p<0.001, **** p<0.0001.

Supplementary Figure 6. Abundance of autophagy marker proteins stratified by sex.

Levels of (**a**) LC3A-II/LC3A-I ratio, (**b**) total LC3A, (**c**) LC3B-II/LC3B-I ratio, (**d**) total LC3B, (**e**) ATG3, (**f)** ATG5, (**g**) ATG7, (**h**) ATG12, (**i**) Beclin-1, and (**j**) SQSTM1/p62 were determined by western blot. Sample size was 5 male and 5 female 18mo controls, 4-5 male and 6 female APP 6→18mo, 4-5 male and 5 female 24mo controls, and 5 male and 5-6 female APP 12→24mo. Individual data points and group means ± S.E.M. are presented. * p<0.05, ** p<0.01, *** p<0.001, **** p<0.0001.

Supplementary Figure 7. Analyses of canonical pathways, diseases and functions, and upstream regulators.

(**a**) Canonical pathways, (**b**) disease and function annotations, and (**c**) upstream regulators associated with differentially expressed genes were identified by comparing brain transcriptomic profiles between APP 6→18mo (2 males and 2 females), APP 12→24mo (2 males and 2 females), and age-matched controls (2 male and 1 female 18-month-old controls; 2 male and 2 female 24-month-old controls) using Ingenuity Pathway Analysis (IPA). A false discovery rate (FDR) of 0.05 was applied. Predicted pathway activation is shown in red, while predicted inhibition is indicated in blue.

Supplementary Figure 8. Gene expression levels of astrocytic and microglial lineage markers determined by qPCR.

mRNA levels of (**a**) *Gfap*, (**b**) *Itgam*, (**c**) *Trem2*, and (**d**) *Cd68* were quantified in brain tissue from APP 6→18mo (4 males and 4 females), APP 12→24mo (4 males and 4 females), and age-matched control animals (4 male and 4 female 18mo controls; 4 male and 3-4 female 24mo controls). Individual data points as well as means ± S.E.M. are presented. * $p<0.05$, ** $p<0.01$, *** $p<0.001$, **** $p<0.0001$.

Supplementary Figure 9. Protein abundance of astrocytic and microglial lineage markers measured by western blot.

(**a**) Representative western blot images and quantification of (**b**) GFAP, (**c**) CD11b, (**d**) CD68, (**e**) IBA1, and (**f**) TREM2 in 18mo control (5 males and 5 females), APP 6→18mo (5 males and 6 females), 24mo control (5 males and 5 females), and APP 12→24mo (5 males and 6 females) are shown. Individual data points as well as means ± S.E.M. are presented. * $p<0.05$, ** $p<0.01$, *** $p<0.001$, **** $p<0.0001$.

Supplementary Figure 10. Sex-stratified analyses of cholinergic markers at the transcriptional and protein levels.

mRNA levels of (**a**) *Chat*, (**b**) *Slc5a7*, and (**c**) *Slc18a3* were measured by qPCR in 18mo control (4 males and 4 females), APP 6→18mo (4 males and 4 females), 24mo control (4 males and 3-4 females), and APP 12→24mo (4 males and 3-4 females). Protein levels of (**d**) ChAT and (**e**) SLC5A7 were quantified by western blot in 5 male and 5 female 18mo controls, 5 male and 5-6 female APP 6→18mo, 4-5 male and 5 female 24mo controls, and 4-5 male and 6 female

APP 12→24mo. Individual data points and group means ± S.E.M. are presented. * $p<0.05$, ** $p<0.01$, *** $p<0.001$.

Supplementary Figure 11. Abundance of synaptic marker proteins was reduced with age but was not affected by APP expression.

(**a**) Representative western blot images are shown. Protein levels of (**b**) PSD95 and (**c**) synaptophysin (SYP) were quantified by western blot in 18mo control (5 males and 5 females), APP 6→18mo (5 males and 6 females), 24mo control (4-5 males and 5 females), and APP 12 →24mo (5 males and 6 females). Individual data points as well as means ± S.E.M. are presented. * $p<0.05$, ** $p<0.01$.

Supplementary Data 1. Differentially expressed genes identified by whole-brain RNA sequencing.

This file contains genes differentially expressed in association with sex, age, mutant APP expression, and the age × APP interaction. Analyses were performed by comparing brain transcriptomes of APP 6→18mo (2 males and 2 females), APP 12→24mo (2 males and 2 females), and age-matched control mice (2 male and 1 female 18-month-old controls; 2 male and 2 female 24-month-old controls). A false discovery rate (FDR) threshold of 0.05 was applied.

Supplementary Data 2. Canonical pathway, disease/function, and upstream regulator analyses by Ingenuity Pathway Analysis.

This file summarizes canonical pathways, disease and biological functions, and upstream regulators identified through Ingenuity Pathway Analysis (IPA) of differentially expressed genes in association with age, mutant APP expression, and the age × APP interaction. Analyses were based on comparisons of brain transcriptomic profiles between APP 6→18mo (2 males and 2 females), APP 12→24mo (2 males and 2 females), and age-matched control mice (2 male and 1 female 18-month-old controls; 2 male and 2 female 24-month-old controls). A false discovery rate (FDR) threshold of 0.05 was applied.
